# Tertiary lymphoid structure stratifies glioma into three distinct tumor subtypes

**DOI:** 10.18632/aging.203798

**Published:** 2021-12-26

**Authors:** Xingwang Zhou, Wenyan Li, Jie Yang, Xiaolan Qi, Yimin Chen, Hua Yang, Liangzhao Chu

**Affiliations:** 1 Department of Neurosurgery, The Affiliated Hospital of Guizhou Medical University, Guiyang 550004, Guizhou Province, PR China; 2 Key Laboratory of Endemic and Ethnic Diseases, Ministry of Education and Key Laboratory of Medical Molecular Biology of Guizhou Province, Guizhou Medical University, Guiyang 550004, Guizhou Province, PR China

**Keywords:** tertiary lymphoid structure, glioma, TCGA, CGGA

## Abstract

Objective: Tertiary lymphoid structure (TLS), also known as ectopic lymphoid organs, are found in cancer, chronic inflammation, and autoimmune diseases. However, the heterogeneity of TLS in gliomas is unclear. Therefore, it is necessary to identify TLS differences and define TLS subtypes.

Methods: The TLS gene profile of 697 gliomas from The Cancer Genome Atlas (TCGA) was used for consensus clustering to identify robust clusters, and the reproducibility of the stratification method was assessed in Chinese Glioma Genome Atlas (CGGA) cohort1, CGGA_cohort2, and GSE16011. Analyses of clinical characteristics, immune infiltration, and potential biological functions were performed for each subtype.

Results: Three resulting clusters (A, B, and C) were identified based on consensus clustering on the gene expression profile of TLS genes. There was a significant prognostic difference among the clusters, with a shorter survival for C than B and A. In comparison with the A and B subtypes, the C subtype was significantly enriched in primary immunodeficiency, intestinal immune network for lgG production, antigen processing and presentation, natural killer cell-mediated cytotoxicity, complement and coagulation cascades, cytokine-cytokine receptor interaction, leukocyte transendothelial migration, and some immune-related diseases. The levels of 23 immune cell types were higher in the C subtype than in the A and B subtypes. Finally, we developed and validated a riskscore based on TLS subtypes with better performance of prognosis prediction.

Conclusions: This study presents a new stratification method according to the TLS gene profile and highlights TLS heterogeneity in gliomas.

## INTRODUCTION

Gliomas are common malignant tumors in the central nervous system [1]. Although glioma patients receive tumor resection following chemotherapy and radiotherapy [2], as well as tumor-treating fields [3], their prognosis remains poor. As a result, understanding the biological mechanism of glioma progression is crucial for glioma therapy. Immunotherapy, which is used to modulate lymphocytes to attack tumor cells and prevent tumor progression, has attracted considerable attention [4]. However, the efficacy of immunotherapy for glioblastoma patients is limited [5]. Therefore, it is important to further analyze potential resistance factors and develop new treatment strategies.

Tertiary lymphoid structure (TLS), also known as ectopic lymphoid organs, are found in cancer, chronic inflammation, and autoimmune diseases [6]. The composition in cancer-associated TLSs includes B cells, follicular dendritic cells (FDCs), plasma cells, T cells, neutrophils, macrophages, and high endothelial venules (HEVs) [6]. B cell follicles with germinal center characteristics and a T cell-rich zone with mature dendritic cells (DCs) are surrounded by plasma cells. HEVs are found in the vicinity of TLSs, which can mediate the entry of lymphocytes into TLSs [7]. The presence of TLSs has been reported in various tumors including head and neck squamous cell carcinoma [8], lung cancer [9], sarcomas [10], and even gliomas [11]. Furthermore, the presence of TLS has been found to be associated with a good prognosis in the majority of cancers [8–10, 12, 13] and improve the effectiveness of immunotherapy, indicating that TLSs may generate anti-tumorigenic immune cells, which play a vital role in the immune response against tumors. However, some studies indicated that TLS presence may lead to tumor progression, which may be associated with microniches for cancer progenitor cells. Thus far, only one study has evaluated the distribution of TLSs in gliomas [11], and the results showed that αCD40 enhanced the formation of TLSs via the stimulation of B cells while reducing CD8+ T cell cytotoxicity in the brain of glioma-bearing mice [11]. Therefore, it is crucial to determine the TLS profile and identify TLS subtypes in gliomas, which may contribute to the development of new treatment methods.

In the present study, we stratified gliomas into three subtypes according to the unsupervised clustering of TLS signature expression profiles. Three independent cohorts were used to verify the reproducibility and stability of this classification method. Each of the three TLS subtypes had distinct clinical characteristics, biological functions, and immune infiltration patterns. Our findings shed light on TLS heterogeneity in gliomas, and the clinical stratification of TLS may contribute to the development of TLS-targeted therapy.

## RESULTS

### Differential expression and survival analysis

**Figure 1:**
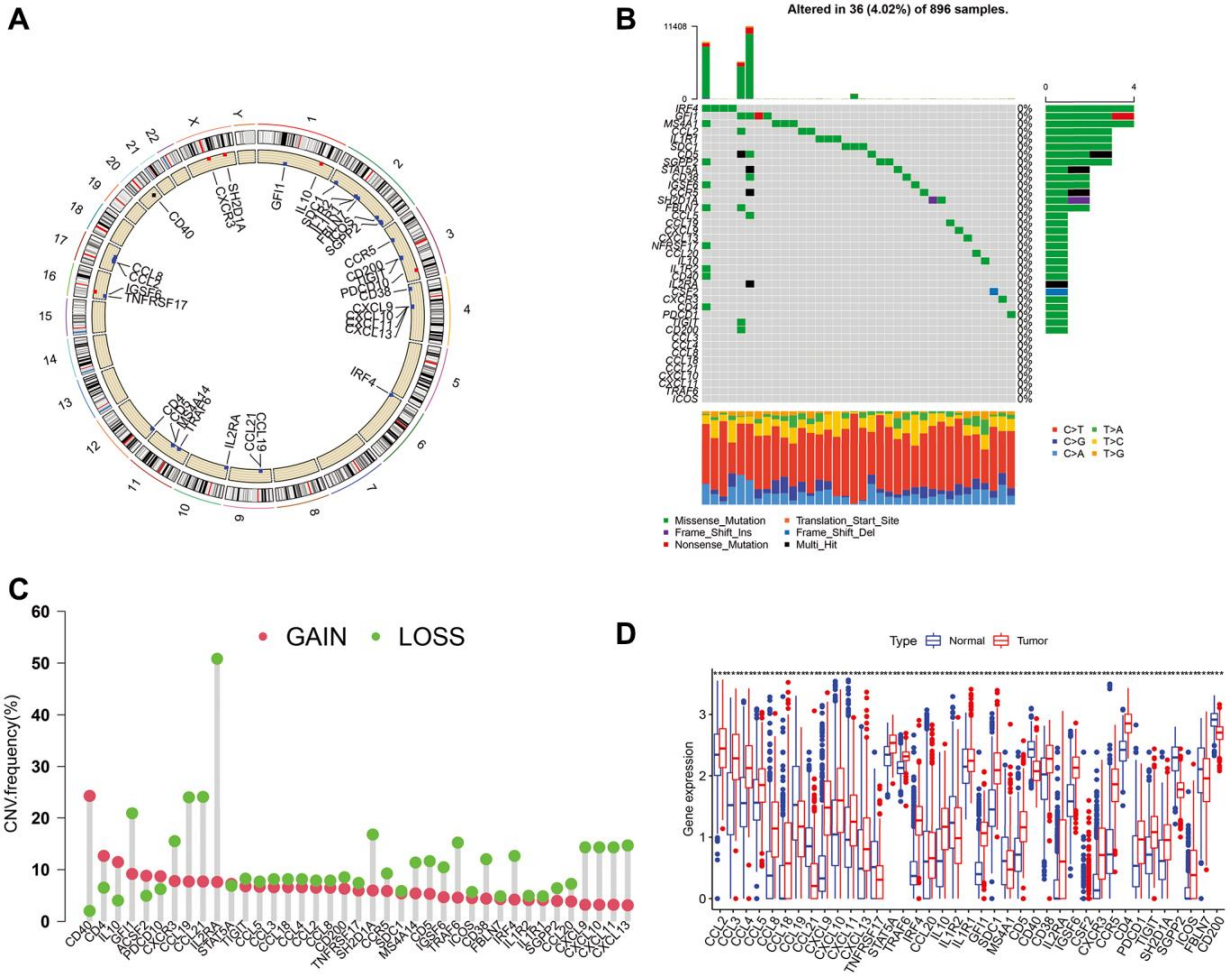
**Mutations, CNVs, and diffent expresion of TLS genes in TCGA cohort.** (**A**) The location of CNVs of TLS genes on 23 chromosomes. (**B**) The somatic mutation frequency of TLS genes in TCGA cohort. (**C**) The CNV frequency of TLS genes. (**D**) The different expresion of TLS between 697 gliomas and 1157 normal brain tissues.

**Figure 2:**
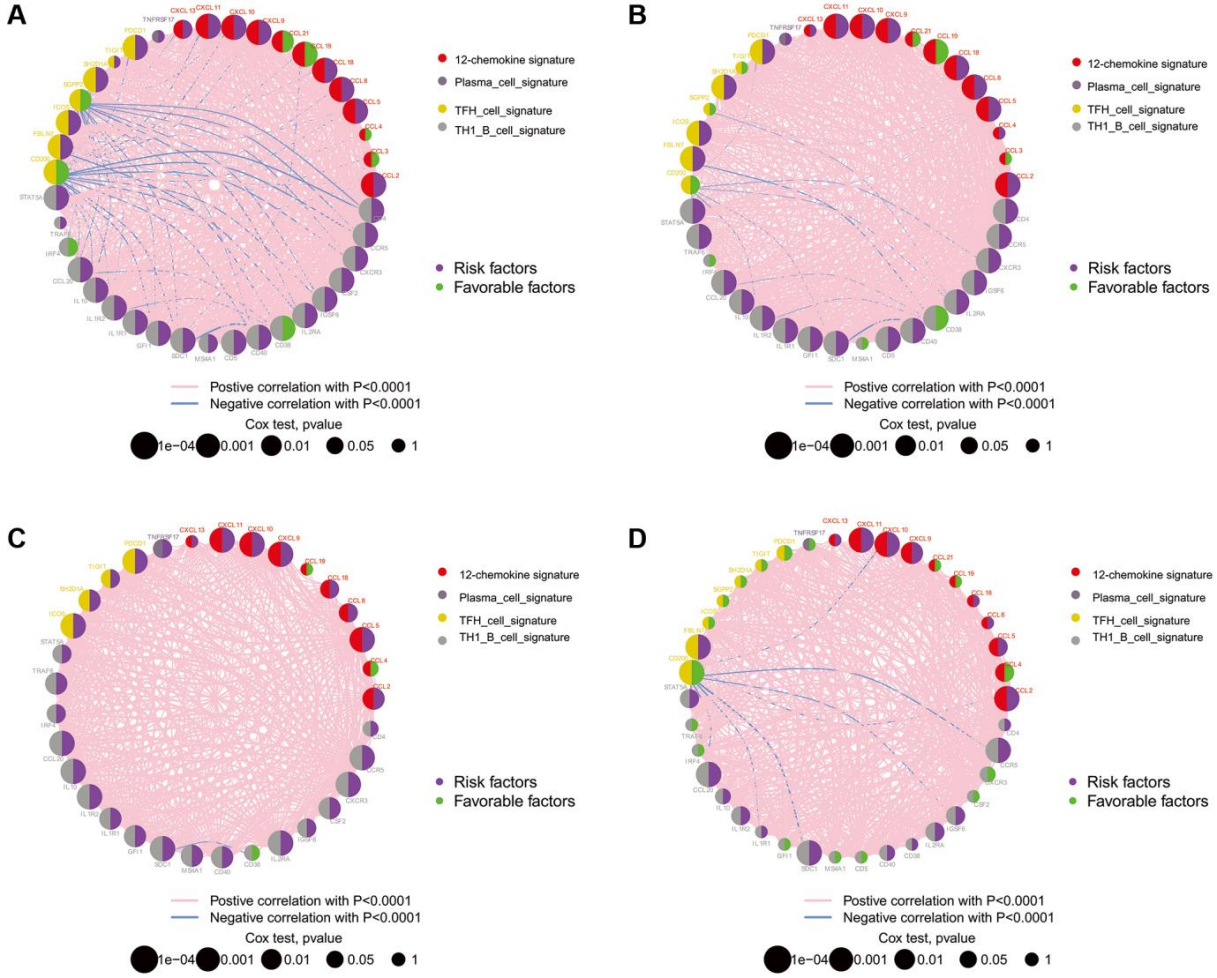
**The prognostic effect of TLS genes in glioma of TCGA, CGGA and GSE16011.** (**A**) TCGA; (**B**) CGGA_cohort1; (**C**) CGGA_cohort2; (**D**) GSE16011. All of the TLS genes were associated with prognosis of glioma patients in these four datasets.

The locations of CNVs and CNV frequency in TLS genes were determined (Figure 1A and 1C). Among 896 glioma patients, only 36 (4.02%) patients harbored somatic mutations in TLS genes (Figure 1B). We evaluated the gene expression profile of TLS genes in glioma and normal tissues (TCGA vs. GTEx). We found that the expression levels of CXCL2, CCL3, CCL4, CCL5, CCL8, CCL18, CXCL9, CXCL8, CXCL11, CXCL13, CD4, CCR5, CXCR3, CSF2, IGSF6, IL2RA, CD38, CD5, SDC1, GFI1, IL1R1, IL10, CCL20, IRF4, TRAF6, STAT5A, ICOS, SH2D1A, TIGIT, PDCD1 were higher in glioma tissues than in normal tissues (Figure 1D, *p* < 0.001); however, CXCL19, CXCL21, TNFRSF17, IL1R2, MS4A1, CD40, SGPP2, CD200, and FBLN7 were downregulated in glioma tissues (*p* < 0.001). Survival analysis indicated that 36 of these 40 genes were associated with the prognosis of glioma in the TCGA cohort (Figure 2A, *p* < 0.05), the genes associated with the prognosis of glioma were identified in CGGA_cohort1, CGGA_cohort2, and GSE16011 (Figure 2B–2D).

### Identification of three subtypes in gliomas with consensus clustering

**Figure 3:**
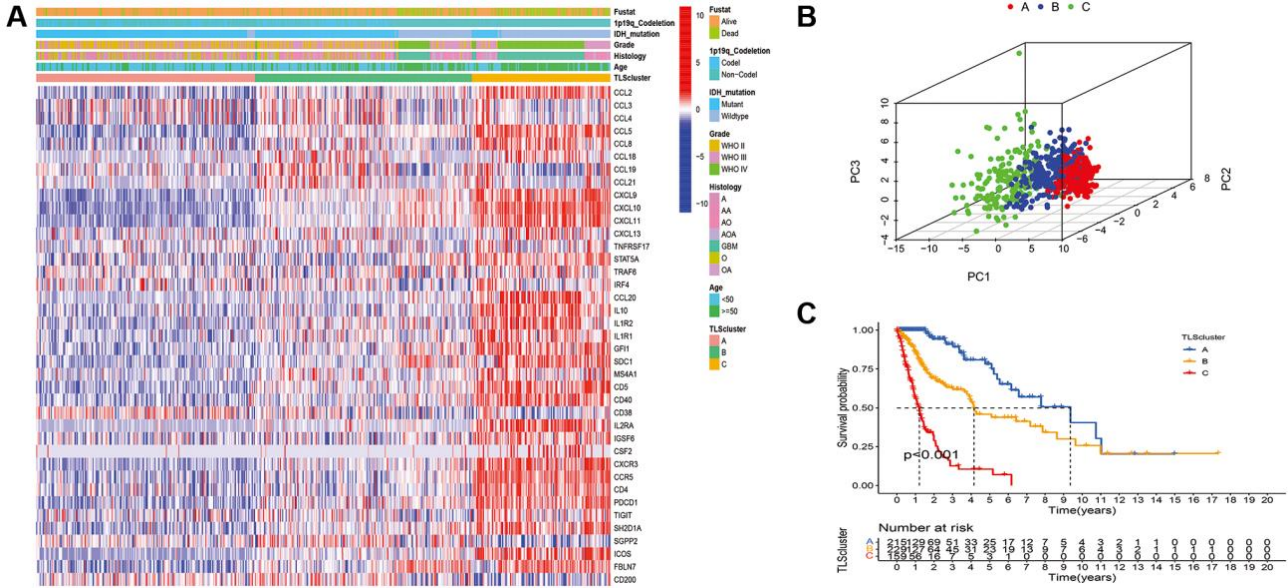
**Identification of distinct TLS subtypes in glioma through TLS gene profiling.** (**A**) Heatmaps of three TLS subtypes defined in TCGA cohorts and the relation between TLS subtypes and clinical features. (**B**) Principal component analysis (PCA) of three metabolic subtypes using candidate genes. (**C**) Survival analyses show significant differences between the three TLS subtypes in TCGA cohorts.

To characterize TLS heterogeneity in gliomas, the 40 TLS genes were used to perform clustering analysis (Supplementary Figure 1). Three clusters (A, B, and C) were identified based on consensus clustering on the gene expression profile of TLS genes (Figure 3A). PCA was carried out to confirm the assignments of subtypes and validate the differences in expression characteristics among the three TLS subtypes (Figure 3B). There was a significant prognostic difference among the clusters, with a shorter survival for C than B and A (Figure 3C, *p* < 0.001).

**Figure 4:**
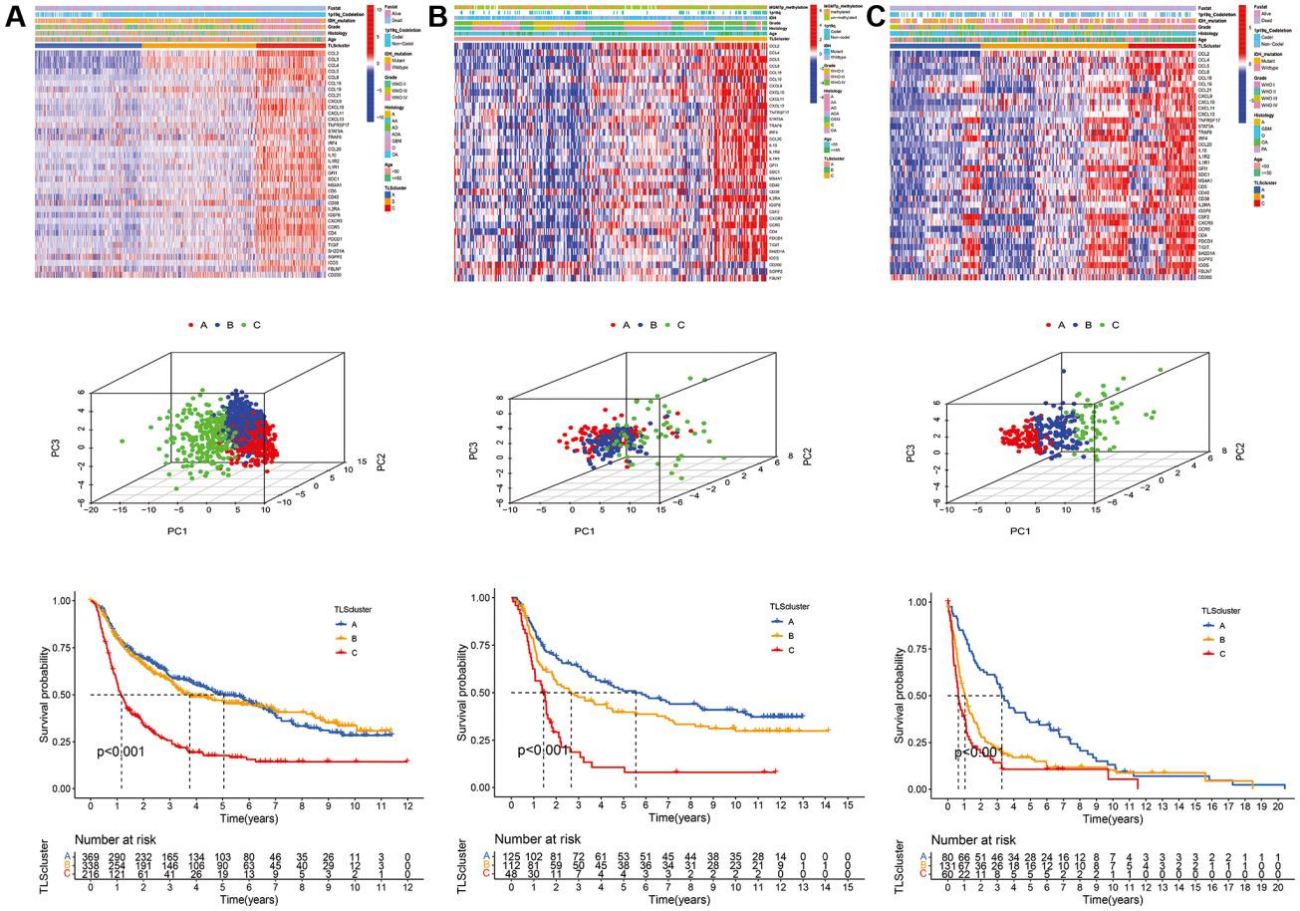
**Validation of TLS subtypes in CGGA_cohort1, CGGA_cohort2 and GSE16011.** (**A**) Heatmap of three TLS subtypes defined in CGGA_cohort1 cohorts and the relation between TLS subtypes and clinical features, PCA of three metabolic subtypes using candidate genes, survival analyses show significant differences between the three TLS subtypes in CGGA_cohort1. (**B**) heatmaps, PCA and survival analyses in CGGA_cohort2. (**C**) heatmaps, PCA and survival analyses in GSE16011.

CGGA_cohort1, CGGA_cohort2, and GSE16011 were used to verify the reproducibility and stability of this classification method in the TCGA cohort (Figure 4). The results showed high consistency between the subtypes of the TCGA cohort and CGGA_cohort1, CGGA_cohort2, and GSE16011 (Supplementary Table 1). In addition, the TLS subtypes of the testing cohorts showed a similar pattern of expression and prognostic characteristics to the TCGA cohort (Figure 4).

### Relationship between TLS subtypes and clinical features

We evaluated the clinical relevance of the identified TLS subtypes. The results indicated that WHO grade III, WHO grade IV, IDH wild-type, 1p19q non-codeletion, glioblastoma, and anaplastic glioma were associated with the C subtype (Figure 3). However, the A subtype was associated with WHO grade II, IDH mutation, 1p19q codeletion, astrocytoma, oligodendroglioma, and oligodendroastrocytoma (Figure 3 and Supplementary Table 2). Similarly, the relationship of TLS subtypes with clinicopathological characteristics was observed in CGGA_cohort1, CGGA_cohort2, and GSE16011 (Figure 4, Supplementary Tables 3–5). Furthermore, we also observed that most classical, neural, proneural gliomas were A subtype, while most mesenchymal gliomas was C subtype (Supplementary Tables 2 and 4).

### Potential biological functions related to TLS subtypes

GSEA was used to identify potential biological functions related to the TLS subtypes. In comparison with the A and B subtypes, the C subtype was significantly enriched in primary immunodeficiency, intestinal immune network for lgG production, antigen processing and presentation, natural killer cell-mediated cytotoxicity, complement, and coagulation cascades, cytokine-cytokine receptor interaction, leukocyte transendothelial migration, and some immune-related diseases such as asthma and systemic lupus erythematosus (Supplementary Figures 2 and 3). In comparison with the A subtype, the B subtype was significantly enriched in cytokine-cytokine receptor interaction, natural killer cell-mediated cytotoxicity, primary immunodeficiency, intestinal immune network for lgG production, antigen processing and presentation, and some immune-related diseases such as asthma and systemic lupus erythematosus (Supplementary Figure 4). Similar results were obtained for CGGA_cohort1, CGGA_cohort2, and GSE16011 (Supplementary Figures 5–13).

### Immune infiltration of TLS subtypes in gliomas

**Figure 5:**
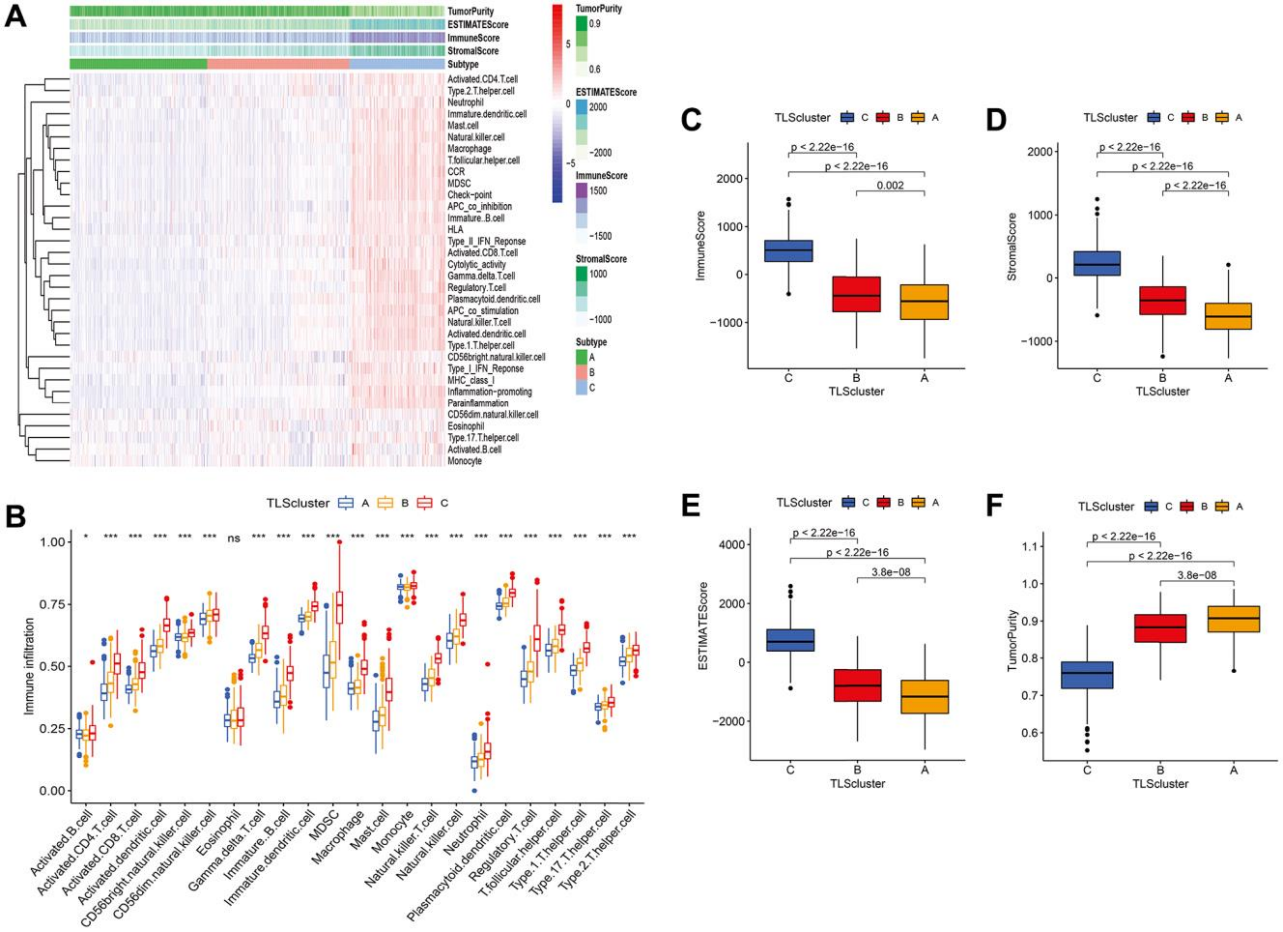
**Immune infiltration and tumor microenvironment of three TLS subtypes in TCGA cohort.** (**A**) Heatmap of TLS subtypes associated with immune infiltration and immune funtion. (**B**) the signature of 23 immune cell among TLS subtypes. (**C**–**F**) tumor microenvironment of TLS subtypes. (**C**) subtype had higher immune, stromal, and ESTIMATE scores compared with the scores of the A and B subtypes; however, tumor purity was lower (Figure 5C–5F).

We used ssGSEA to determine the functions and enrichment levels of immune cells. The levels of 23 immune cell types were higher in the C subtype than in the A and B subtypes, such as activated B cells, activated CD4 T cells, activated CD8 T cells, and activated DCs (Figure 5A and 5B). In terms of immune function, APC co-inhibition, APC co-stimulation, CCR, immune checkpoint, cytolytic activity, HLA, inflammation promotion, MHC class I, parainflammation, type I IFN response, and type II IFN response were significantly enriched in the C subtype compared with the A and B subtypes (Figure 5B and 5C). In addition, we found that the C subtype had higher immune, stromal, and ESTIMATE scores compared with the scores of the A and B subtypes (Figure 5D and 5E); however, tumor purity was lower (Figure 5F). To validate the results, we quantified the immune infiltration, immune, stromal, and ESTIMATE scores of each subtype in GSE16011, CGGA_cohort1, and CGGA_cohort2, and consistent results were obtained (Supplementary Figures 14–17).

### The potential therapeutic value of TLS subtype

**Figure 6:**
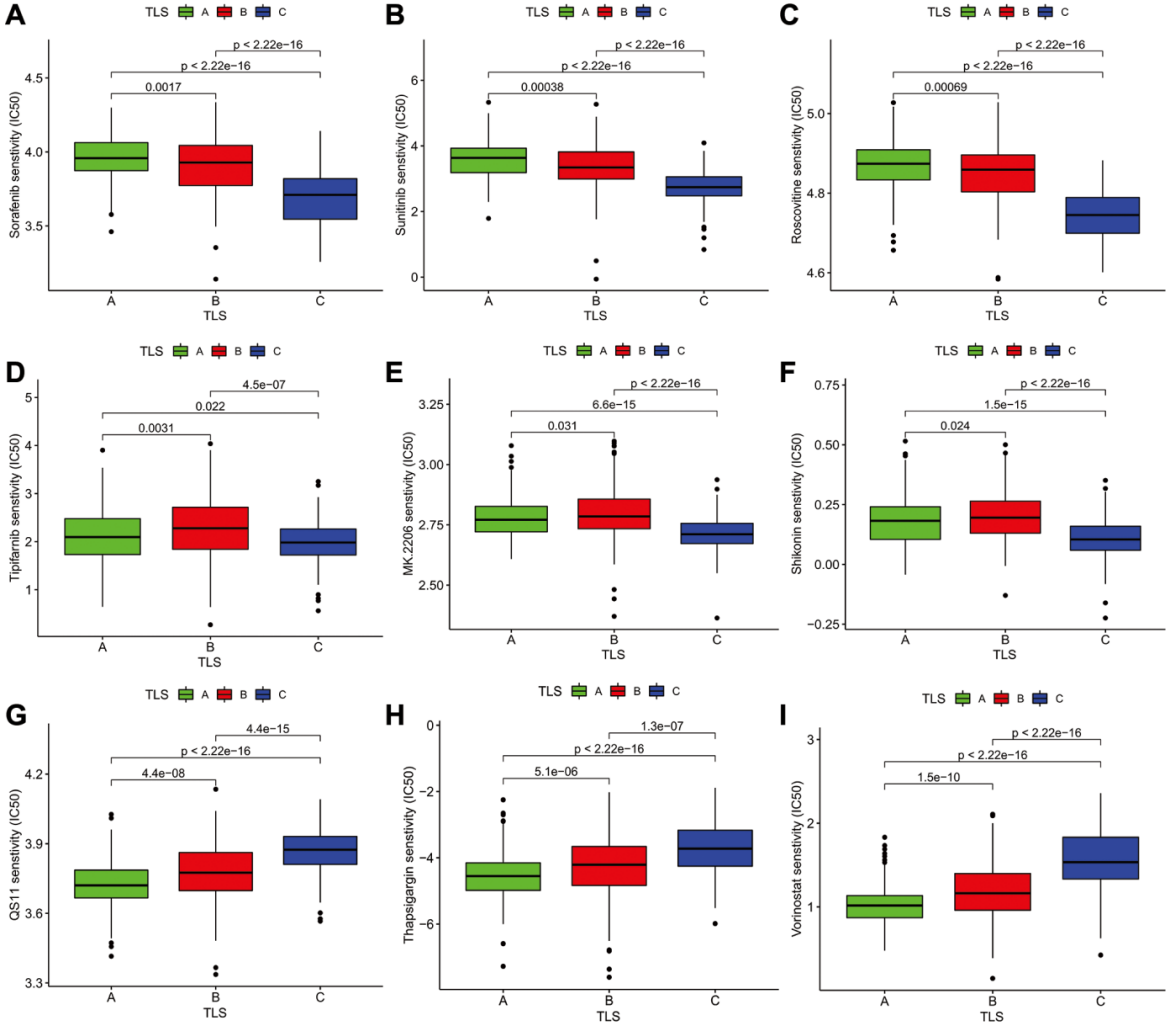
**The Association analysis of TLS subtype and drug sensitivity.** (**A–C**) Sorafenib, Sunitinib, Roscovitine were the most sensitive drugs to A subtype. (**D–F**) Tipifarnib, MK.2206, and Shikonin were the most sensitive drugs to B subtype. (**G–I**) QS11, Thapsigargin, and Vorinostat were the most sensitive drugs to C subtype.

To further understand the effect of the TLS subtype on the drug response, we evaluate the relationship between distinct TLS subtypes and drug sensitivity. We found that drug sensitivity associated with C subtype, including AG.014699, BAY.61.360, BIRB.0796, BMS.754807, CCT007093, EHT.1864, Elesclomol, FH535, GW.441756, Imatinib, Lenalidomide, LFM.A13, OSI.906, PD.173074, PD.0332991, PF.562271, QS11, Thapsigargin, Vinorelbine, Vorinostat, VX.702, ABT.263, AICAR, AZD.0530, AZD8055, BMS.708163, Gefitinib (Supplementary Table 6). The drug sensitivity correlated with B subtype including AP.24534, AS601245, ATRA, and so on. However, many drugs showed a high sensitivity for the A subtype, such as Parthenolide, Paclitaxel, and Temsirolimus (Figure 6 and Supplementary Table 6). All in all, these results indicate that TLS correlated with drug sensitivity. Thus, the TLS may be a potential biomarker for establishing appropriate treatment strategies.

### Calculation of riskscores and validation

**Figure 7:**
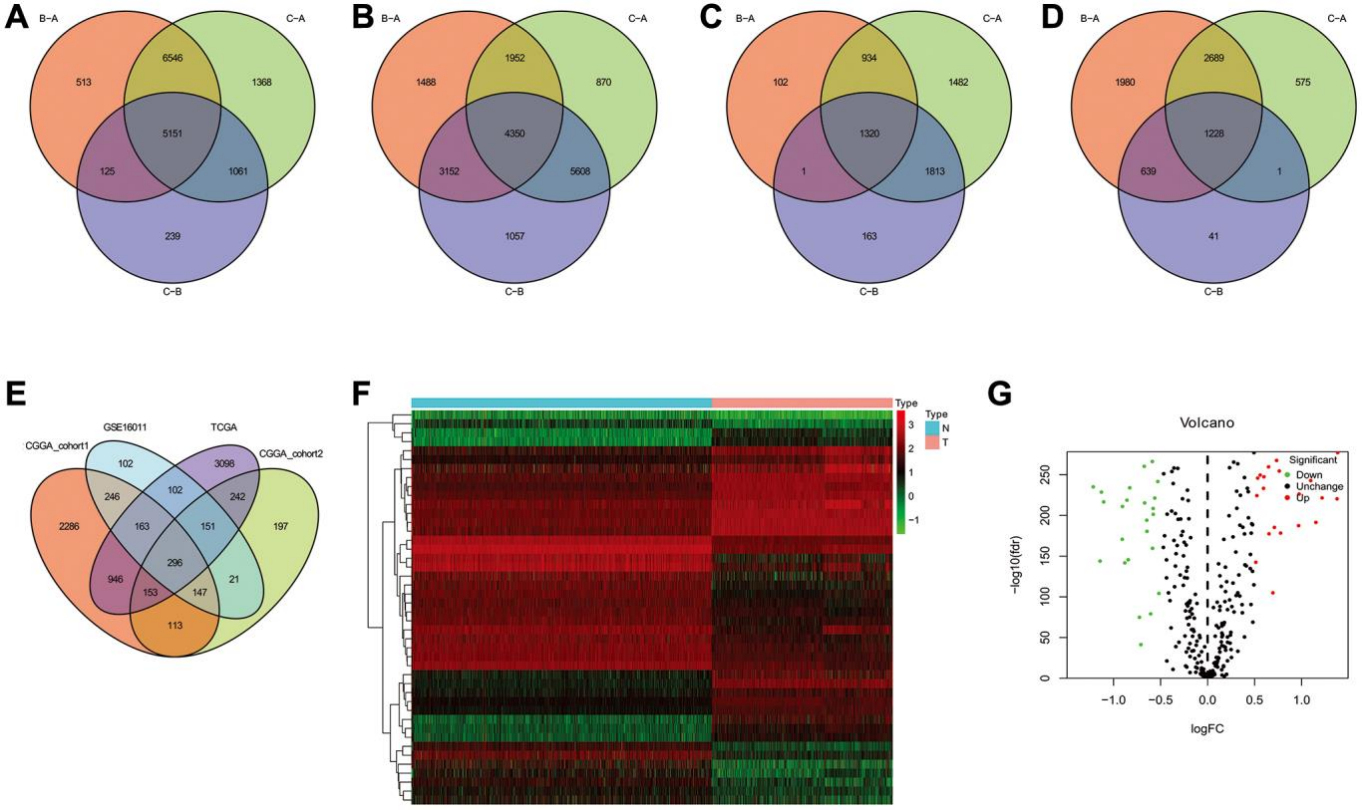
**The common genes among TLS subtypes from TCGA, CGGA_cohort1, CGGA_cohort2 and GSE16011.** (**A**–**D**) the intersection genes of TLS subtypes observed from TCGA, CGGA_cohort1, CGGA_cohort2 and GSE16011, respectively. (**E**) the common genes among TCGA, CGGA_cohort1, CGGA_cohort2 and GSE16011. (**F**) the heatmap of different expresion of intersection genes between glioma and normal tissues. (**G**) volcanic map showed the up- and down-regulated common genes.

**Figure 8:**
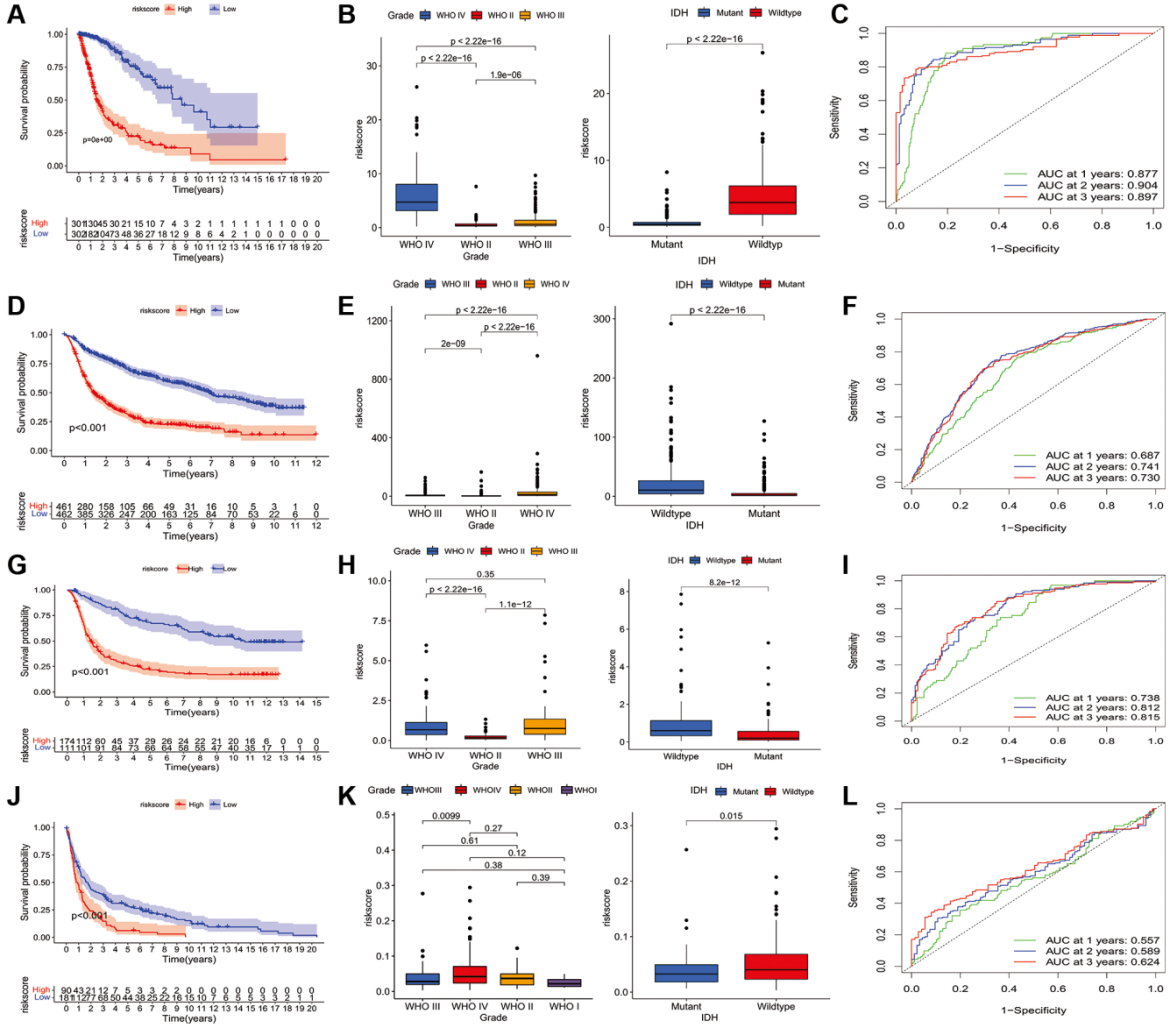
**Construction of riskscore in TCGA and validation in CGGA and GSE16011 dataset.** (**A**) The prognostic role of riskscore in TCGA glioma. (**B**) The relationship between riskscore and tumor grade, and IDH mutation status in TCGA database. (**C**) receiver operating characteristic curve indicated the survival prediction of riskscore in TCGA database; (**D**) The prognostic role of riskscore in glioma of CGGA1. (**E**) The relationship between riskscore and tumor grade, and IDH mutation status of CGGA1 cohort. (**F**) receiver operating characteristic curve indicated the survival prediction of riskscore in CGGA1 database. (**G**) The prognostic role of riskscore in glioma of CGGA2. (**H**) The relationship between riskscore and tumor grade, and IDH mutation status of CGGA2 cohort. (**I**) receiver operating characteristic curve indicated the survival prediction of riskscore in CGGA2 database. (**J**) The prognostic role of riskscore in glioma of GSE16011. (**K**) The relationship between riskscore and tumor grade, and IDH mutation status of GSE16011 cohort. (**L**) receiver operating characteristic curve indicated the survival prediction of riskscore in GSE16011 database. TCGA cohort was used as a discovery set, two CGGA cohorts and GSE16011 were employed as validation sets.

To further analyze the characteristics of the three subtypes, genes with differential expression within subtypes were enquired with the R package “samr”. There were 5151 intersection genes between the A and B subtypes, A and C subtypes, and B and C subtypes in the TCGA cohort (Figure 7A). The intersection genes between subtypes in CGGA_cohort1 (Figure 7B), CGGA_cohort2 (Figure 7C), and GSE16011 (Figure 7D) were identified. The intersection genes from TCGA, CGGA_cohort1, CGGA_cohort2, and GSE16011 were then analyzed again using the R package “Venn” (Figure 7E). Finally, a total of 296 genes were identified. Among these genes, only 44 genes were differentially expressed (24 downregulated and 20 upregulated) between glioma and normal tissues (Figure 7F and 7G). Of the 44 genes, 28 genes were associated with prognosis and selected for LASSO regression analysis to identify the best genes for calculating riskscores in the TCGA dataset. Finally, 14 genes (HAMP, CARD16, TRIM38, CCR5, S100A8, MSR1, S100A9, S100A4, CHI3L2, PLAU, GCH1, P2RY8, UPP1, PROS1) were obtained, and riskscores were calculated with regression coefficients. Survival analysis indicated that patients with high riskscores had shorter overall survival compared with that of patients with low riskscores (Figure 8A). Moreover, multivariate Cox regression analysis suggested that the riskscore was an independent factor in predicting the prognosis of gliomas (Supplementary Figure 17). Analysis of clinical relevance revealed that a high riskscore was related to gliomas with WHO grade IV or IDH wild-type (Figure 8B). The AUCs of riskscores in predicting the 1-year, 3-year, and 5-year survival were 0.877, 0.904, and 0.897, respectively (Figure 8C). We validated this TLS signature in CGGA_cohort1, CGGA_cohort2 and GSE16011, and consistent results were obtained (Figure 8D–8L).

### The clinical significance of intersection proteins between subtypes in glioma confirmed by immunohistochemistry

**Figure 9:**
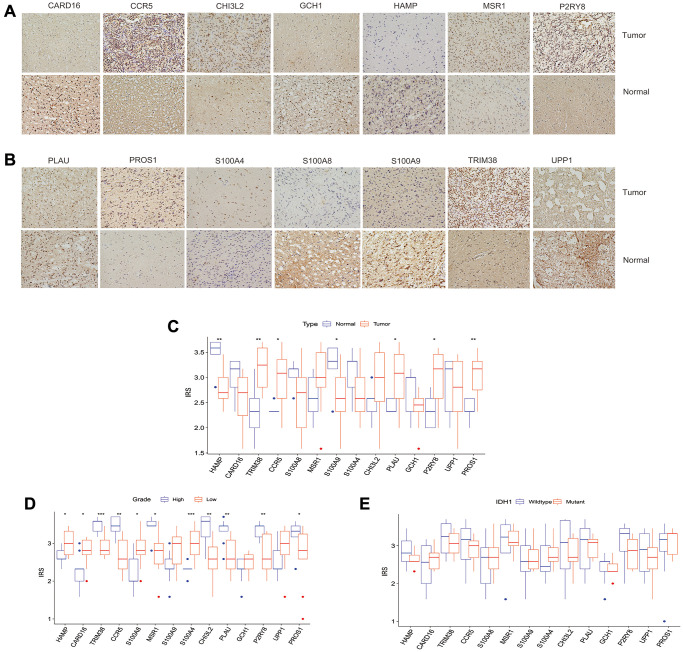
**The clinical significance of intersection proteins between TLS subtypes in glioma confirmed by immunohistochemistry.** (**A** and **B**) The typical image showed the expression status of the 14 intersection proteins in glioma and normal tissues. (**C**) The expression status of the 14 intersection proteins between glioma and normal tissues. (**D**) The expression of the 14 proteins associated with tumor grade. (**E**) the expression of the 14 proteins associated with IDH1 mutation status.

We used immunohistochemistry to evaluate the expression of the above-mentioned 14 genes (HAMP, CARD16, TRIM38, CCR5, S100A8, MSR1, S100A9, S100A4, CHI3L2, PLAU, GCH1, P2RY8, UPP1, PROS1) in glioma and normal tissues. The results showed that TRIM38, CCR5, PLAU, P2RY8, and PROS1 have a higher immunoreaction score (IRS) in tumors than normal tissues (*p* < 0.05, Figure 9A–9C), while the IRS of HAMP and S100A9 in gliomas were lower than normal tissues (*p* < 0.05, Figure 9A–9C). No significant differences were observed for the expression of the remaining proteins between tumor and normal tissues. Furthermore, we assess the differential expression of the 14 proteins between high and low-grade gliomas. We observed that the IRSs of PROS1, P2RY8, PLAU, CHI3L2, MSR1, CCR5, and TRIM38 were higher than its corresponding IRSs in low-grade glioma, while the IRSs of HAMP, CARD16, and S100A8 in high-grade glioma were lower than low-grade glioma (Figure 9D). However, no one of those 14 proteins were found to be associated with IDH1 mutation (Figure 9E).

## DISCUSSION

Gliomas are one of the most common primary tumors in adult patients, which can lead to high mortality and morbidity due to rapid progression and treatment resistance. TLSs are discrete, structured organizations of infiltrating immune cells, which have been found to improve immunotherapy and survival in several cancers. In the present study, we stratified gliomas into three subtypes according to the unsupervised clustering of TLS signature expression profiles. The relevance of clinical characteristics, immune infiltration, tumor microenvironment, potential biological functions was investigated. The findings may shed light on the molecular subtypes of gliomas and deepen our understanding of TLS heterogeneity in gliomas.

An increasing number of studies have shown that an orchestrated immune response to cancer is elicited locally in TLS, which resemble the structures of secondary lymphoid organs [14]. TLS predominantly consists of B cells and T cells. FDCs and germinal centers (GCs) can be used to determine the maturation of TLS. FDCs but not GCs are enriched in B cells and T cells in the intermediate mature stage; however, both FDCs and GCs are found in mature TLSs. Neither FDCs nor GCs is found in immature TLS [15, 16]. Together with T and B cells, DCs, neutrophils, macrophages, HEVs, CD4+ T follicular helper cells (T_FH_), CD8+ cytotoxic T cells, CD4+ regulatory T cells (T_Reg_), and innate lymphoid cells can be detected in TLS [14]. Several gene signatures have been used to detect TLSs in the transcriptomic analyses of human cancers, which include 12 chemokine signatures, T_FH_ cell signatures, T_H_1 and B cell signatures, and plasma cell signatures [6]. To ensure the successful detection of all TLS, these gene signatures were used to investigate TLS heterogeneity in gliomas. The prognostic role of TLSs has been reported in various cancers. The presence of TLS detected by immunohistochemistry was associated with favorable clinical outcomes for lung metastases in colorectal cancer, liver metastases in colorectal cancer, lung cancer, and metastases in ovarian cancer [8–10, 12, 17]. In addition, some studies based on the 12 chemokine signatures indicated that TLSs may be associated with improved survival prognosis in hepatocellular carcinoma [18] and metastases of melanoma [19]. In the current study, 31 TLS genes were upregulated, whereas the other 9 genes were downregulated, and all of these genes were associated with glioma prognosis. Consistently, a significant survival difference was noted among the subtypes, with a shorter overall survival for the C subtype than the A and B subtypes. These results highlighted the importance of TLS-related genes and TLS heterogeneity in predicting the prognosis of glioma patients.

We investigated the association of TLS subtypes with clinical features in the TCGA cohort and found that malignant characteristics were enriched in the C subtype. However, the A subtype was associated with WHO grade II, IDH mutation, 1p19q codeletion, astrocytoma, oligodendroglioma, and oligodendroastrocytoma. Similarly, the relationship of TLS subtypes with clinicopathological characteristics was observed in CGGA_cohort1, CGGA_cohort2, and GSE16011. Our findings could contribute to a better understanding of the pathology and molecular subtypes of gliomas.

TLSs have been reported to improve the immunotherapy of cancer patients, as they are the primary sites of the initiation and maintenance of the local immune response [20, 21]. The underlying mechanisms may involve the activation and differentiation of B cells into antibody-producing cells, which is consistent with the idea that TLSs could improve the *in situ* production of tumor-specific antibodies to enhance anti-tumor immunity [22, 23]. Furthermore, TLSs may increase the proportions of CD69+ and CD38+ activated T cells and CD8+ T cells with an effector memory phenotype, characterized by the overexpression of a set of genes characteristic of T_H_1 cell skewing, T cell cytotoxicity, T cell activation, and T cell chemotaxis [7, 17, 24–27]. In this study, we also found that the B and C subtypes were significantly enriched in primary immunodeficiency, intestinal immune network for lgG production, antigen processing and presentation, natural killer cell-mediated cytotoxicity, complement, and coagulation cascades, cytokine-cytokine receptor interaction, and leukocyte transendothelial migration. The C subtype have higher immune, stromal, and ESTIMATE scores compared with the scores of the A and B subtypes; however, tumor purity was lower. The levels of all 23 immune cell types were higher in the C subtype than in the A and B subtypes. These results demonstrated that the C subtype was a type of glioma with high immune infiltration but poor prognosis. Anti-tumorigenic immune cells such as natural killer cells, B cells, and CD8+ T cells were increased in C subtype, while pro-tumorigenic immune cells including M2 polarized macrophages, T-helper 2 cells, myeloid-derived suppressor cells, and regulatory T cells also increased, the beneficial effect of TLS may be reduced in patients. Previous studies indicated that immunosuppressive cells were presented in TLS such as regulatory T cells. Furthermore, TLSs have been reported to be associated with increased T cell infiltration [11]. As a result, we evaluated the relationship between distinct TLS subtypes and drug sensitivity to understand the effect of the TLS subtype on the drug response. We screened a variety of sensitive drugs for each TLS subtype. However, whether these drugs play an important role in the treatment of glioma based on the TLS subtype needs to be verified by further studies. Regulating the formation of TLS to promote the immunotherapy effect has become one of the priorities of tumor immunotherapy [28], which suggested that TLS could be exploited therapeutically, in particular for nonresponsive, immune “cold” cancers. However, TLS formation induced by immunostimulatory agonistic CD40 antibodies was observed to impair the efficacy of anti-PD-1 antibodies (αPD-1) in murine gliomas through the accumulation of CD11b-expressing B cells, which may inhibit CD8+ T cell responses [11]. Thus, it is important to identify the potential reagents to improve the TLS formation with increasing anti-tumorigenic immune cells but not pro-tumorigenic immune cells including M2 polarized macrophages, T-helper 2 cells, myeloid-derived suppressor cells, and regulatory T cells.

Last, the calculated riskscore based on the intersection gene among TLS subtype suggested that patients with high riskscores had shorter overall survival compared with that of patients with low riskscore, which further demonstrated the prognostic relevance of TLS signature. The 14 proteins found significantly differentially expressed between TLS clusters. TRIM38, CCR5, PLAU, P2RY8, and PROS1 were upregulated in glioma tissues, while HAMP and S100A9 were downregulated. We observed that PROS1, P2RY8, PLAU, CHI3L2, MSR1, CCR5, TRIM38, HAMP, CARD16, and S100A8 were associated with tumor grade. Further studies with a large sample size should be performed to confirm our results or unearth the corresponding cellular mechanism. The results and conclusions of the present study were primarily obtained via bioinformatics analysis based on TCGA, CGGA, and GSE16011 cohorts. The distribution of TLS and modulation of TLS formation in glioma tissues should be explored in future studies.

In summary, we stratified gliomas into three subtypes according to the unsupervised clustering of TLS signature expression profiles. The relevance of clinical characteristics, immune infiltration, tumor microenvironment, potential biological functions, and drug sensitivity were investigated. The findings may shed light on the molecular subtypes of gliomas and deepen our understanding of TLS heterogeneity in gliomas. The study presents a method of stratification for the therapeutic induction of specific TLSs to enhance anti-cancer immunotherapy.

## METHODS

### Patients and datasets

A total of 2090 glioma patients from public datasets including The Cancer Genome Atlas (TCGA), Chinese Glioma Genome Atlas (CGGA), and GSE16011 were enrolled in this study. For the TCGA dataset (http://cancergenome.nih.gov/), we downloaded the RNA-seq data, copy number alterations (CNAs), somatic mutations, and the corresponding clinical information [29]. The CGGA database included two RNA-seq datasets and one microarray dataset with the corresponding clinical information (http://www.cgga.org.cn). The RNA-seq, microarray datasets, and the corresponding clinical information were extracted [29]. We combined the two RNA-seq datasets and named it CGGA_cohort1, and the microarray dataset was named CGGA_cohort2. For the GSE16011 cohort, we extracted microarray data accompanied with clinical information from the GEO database (https://www.ncbi.nlm.nih.gov/geo/) [29]. The gene expression data of normal brain tissues were extracted from Genotype-Tissue Expression (GTEx, https://gtexportal.org/) [30].

### Construction and validation of TLS subtypes

Recently, Catherine et al. summarized the gene signatures required for TLS analysis identified from the transcriptomic analyses of human cancers, which include 12 chemokine signatures (CCL2, CCL3, CCL4, CCL5, CCL8, CCL18, CCL19, CCL21, CXCL9, CXCL8, CXCL11, CXCL13), T_FH_ cell signatures (CXCL13, CD200, FBLN7, ICOS, SGPP2, SH2D1A, TIGIT, PDCD1), T_H_1 cell and B cell signatures (CD4, CCR5, CXCR3, CSF2, IGSF6, IL2RA, CD38, CD40, CD5, MS4A1, SDC1, GFI1, IL1R1, IL1R2, IL10, CCL20, IRF4, TRAF6, STAT5A), a plasma cell signature (TNFRSF17), and a CXCL13 signature (CXCL13) [6]. To ensure that all TLS signatures can be detected, consensus clustering was performed based on the 12 chemokine signatures, T_FH_ cell signatures, T_H_1 cell and B cell signatures, plasma cell signature, and CXCL13 signature to identify robust clusters of glioma. The optimal k was assessed using a consensus heatmap and the cumulative distribution function. We used the TCGA database as the training cohort to train a partition around medoids (PAM) classifier and validate the TLS subtypes in CGGA_cohort1, CGGA_cohort2, and GSE16011. The reproducibility and similarity of the TLS subtypes among the training and validation cohorts were assessed by the R package “clusterRepro”.

### Gene set variation analysis (GSVA) and functional annotation

The potential biological functions related to TLS subtypes were enriched by gene set variation analysis (GSVA) using the R package “GSVA” [31]. We downloaded the gene set “h.all.v7.2” and “c2.cp.kegg.v7.1” from MsigDB database to perform GSVA analysis. The functional annotation of the TLS related genes was conducted by clusterProfiler R Package [32].

### Calculation of immune cell infiltration for different TLS subtypes

We used ESTIMATE to calculate the stromal score (stromal content), tumor purity, and immune score (immune cell infiltration) for each glioma sample [33]. Single-sample GSEA (ssGSEA) was performed to estimate the infiltration levels of 23 immune signatures by using the R package “GSVA” [31, 34]. The genes were used to define the immune cell were summarized in Supplementary Table 7.

### Association analysis of TLS subtype and drug sensitivity

The R package “pRRophetic” was employed to assess the sensitivity of these three distinct glioma subtypes [35]. The maximum inhibitory concentration (IC50) and prediction accuracy were evaluated by algorithm through 10-fold crossvalidation and ridge regression of the Dependent Cancer Drug Sensitivity Genomics (GDSC) database (https://www.cancerrxgene.org/).

### Calculation of riskscore based on intersection genes among TLS subtypes

Differentially expressed genes (DEGs) were identified by comparing three TLS subtypes, and intersection genes were identified using the R package “Venn”. The intersection genes from TCGA, CGGA_cohort1, CGGA_cohort2, and GSE16011 were then analyzed again using the R package “Venn”. The TCGA database and GTEx were used to evaluate the expression of the intersection genes in tumor and normal tissues. Genes with a false discovery rate (FDR) <0.05 and a log2 fold change (FC) >1 were defined as genes with differential expression [32]. The TCGA database was used to select genes related to prognosis. Prognosis-related genes with differential expression were used to calculate riskscores via LASSO regression in the training cohort of TCGA. Riskscore were then calculated in the testing cohorts including CGGA_cohort1, CGGA_cohort2, and GSE16011 [36]. The prognostic role of the riskscore of glioma patients was assessed in the training and testing cohorts by univariate and multivariate Cox proportional hazards regression analyses.

### Immunohistochemistry

A total of 20 cases of glioma and 5 normal tissues were collected to validate the expression of key proteins found significantly differentially expressed between TLS clusters from our hospital. The clinical information includes gender, age, tumor location, histology, and the common molecule change such as isocitrate dehydrogenase (IDH). The ethical committee of the affiliated hospital of Guizhou medical university approved this study protocol, and informed consent was obtained from patients. The 14 proteins expressed between TLS clusters were assessed by immunohistochemistry staining based on the protocol instruction [37]. Put simply, the thickness of the tissue section is 8 μm. Dewaxing with xylene and rehydration was used to reduce ethanol concentration. Antigen recovery was obtained by boiling slices on 10 mm citrate buffer for 20 minutes. The slices were incubated with background Sniper (Biocare Medical) for 30 minutes at room temperature after the endogenous peroxidase with 3% catalase was blocked in methanol. The sections were then incubated with primary antibodies of the HAMP(1:200, Abcam, #ab31875), CARD16 (1:200, Abcam,#aa1-100), TRIM38 (1:100, Proteintech, #O00635), CCR5(1:200, R&D Systems, #P51681), S100A8(1:250, Abcam,#D3DV36), MSR1 (1:200, Bio-Rad, #E9QNQ5), S100A9 (1:200, Bio-Rad, #D3DV36), S100A4(1:400, Dako, #P20066), CHI3L2 (1:300, GeneTex, #A6NNY3), PLAU(1:100, Proteintech,#P00749), GCH1 (1:200, Abnova, #P30793), P2RY8 (1:500, R&D Systems, #BAA92159), UPP1 (1:300, MilliporeSigma, #Q15362), PROS1 (1:300, Developmental Studies Hybridoma Bank, #P29617) at 4°C overnight with a working concentration of 1:100, respectively. The immunoreaction score (IRS) was used to assess the immunohistochemical staining results. The IRS was defined as the percentage of positive cells Χ staining intensity, which was ranged from 0–12 [38]. The clinical association of IRS for these 14 proteins were evaluated.

### Data analysis

R software (version 4.0.4) was used to perform all analyses. Differences in clinical and molecular features between subtypes were compared by the Chi-square test. One-way ANOVA was carried out for the comparison of three groups. A *p*-value < or = 0.05 was defined as statistically significant.

### Ethics approval and informed consent

The experimental verification of this study was approved by the ethics committee of the affiliated hospital of Guizhou medical university.

## Supplementary Materials

Supplementary Figures

Supplementary Tables 1-5

Supplementary Table 6

Supplementary Table 7
