# Supplementary material for: Tertiary lymphoid structure stratifies glioma into three distinct tumor subtypes
Source: Aging (Albany NY). 2021 Dec 26;13(24):26063–94. doi: 10.18632/aging.203798 (PMC8751592; doi:10.18632/aging.203798)
Supplement: Supplementary Figures [file aging-13-203798-s001.pdf]

SUPPLEMENTARY FIGURES

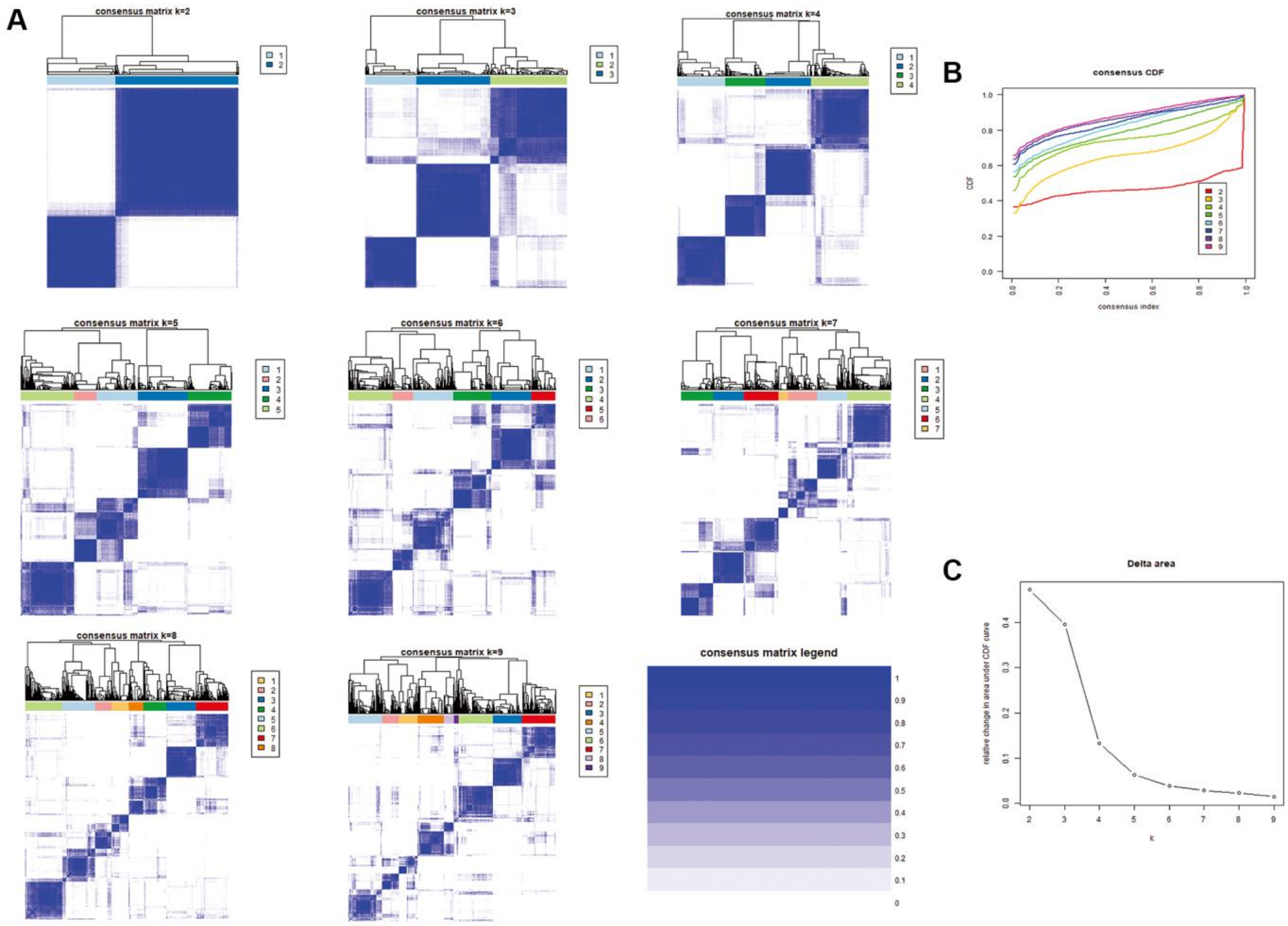

Supplementary Figure 1. Consensus clustering based in TLS gene expression of TCGA glioma. (**A**) Clustering matrix for k = 2 to k = 9. (**B**) CDF (cumulative distribution function) curve for k = 2 to k = 10. (**C**) Relative change in area under CDF curve for k = 2 to k = 10.

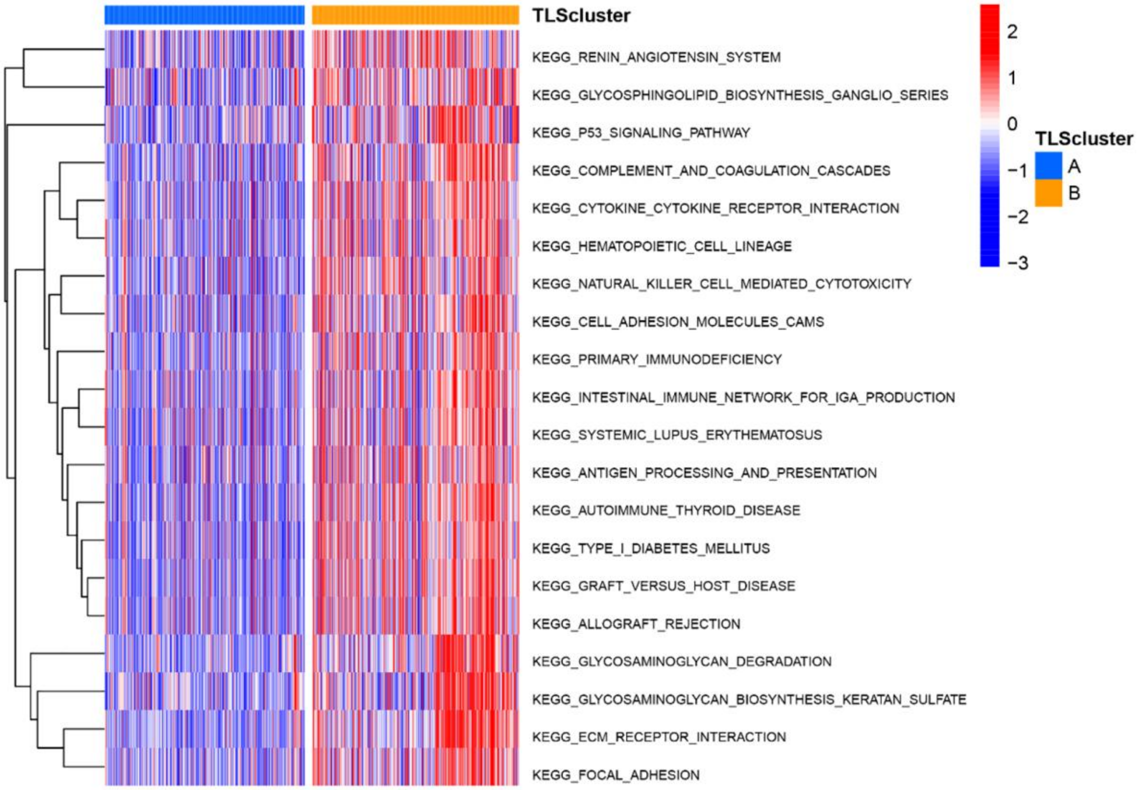

Supplementary Figure 2. The top 20 potential biological functions between subtype A and B in TCGA.

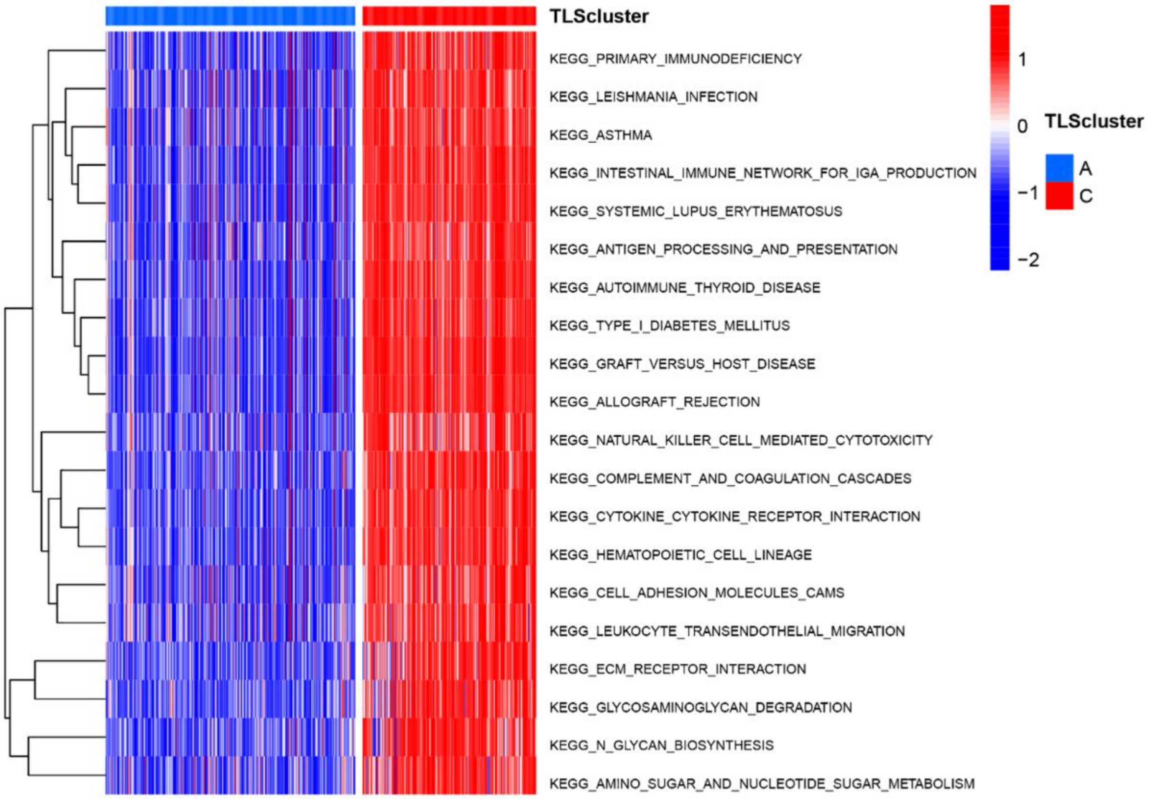

Supplementary Figure 3. The top 20 potential biological functions between subtype A and C in TCGA.

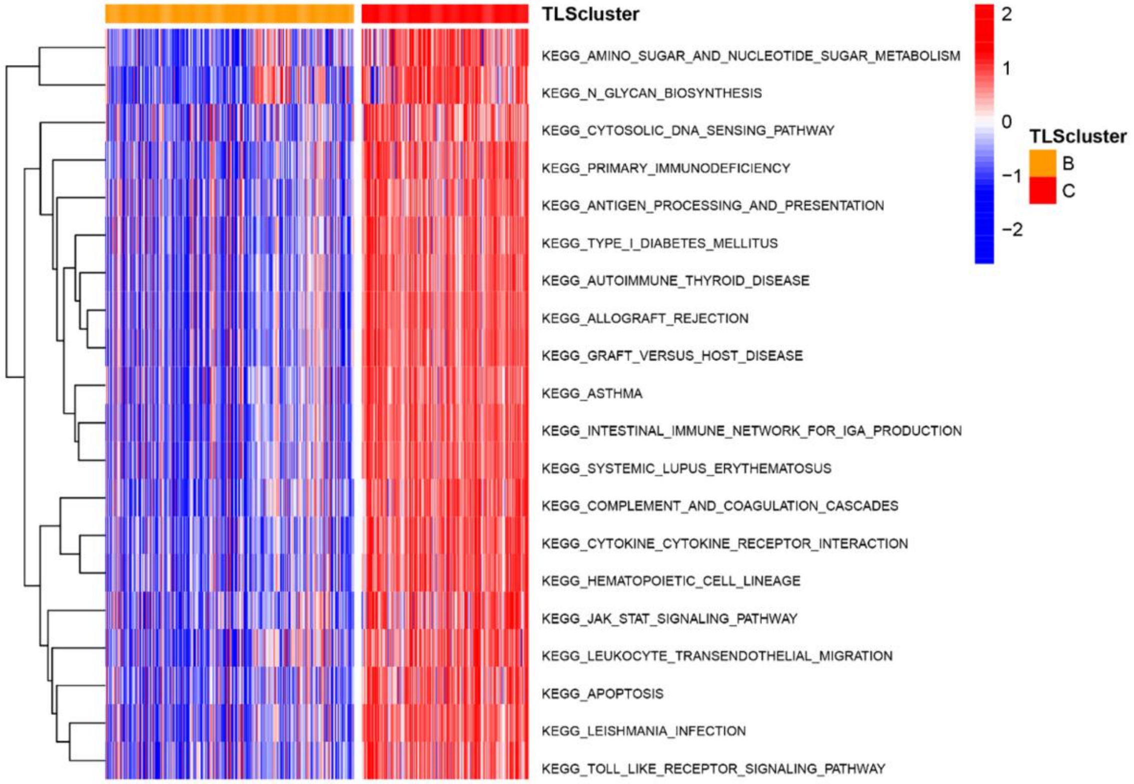

Supplementary Figure 4. The top 20 potential biological functions between subtype B and C in TCGA.

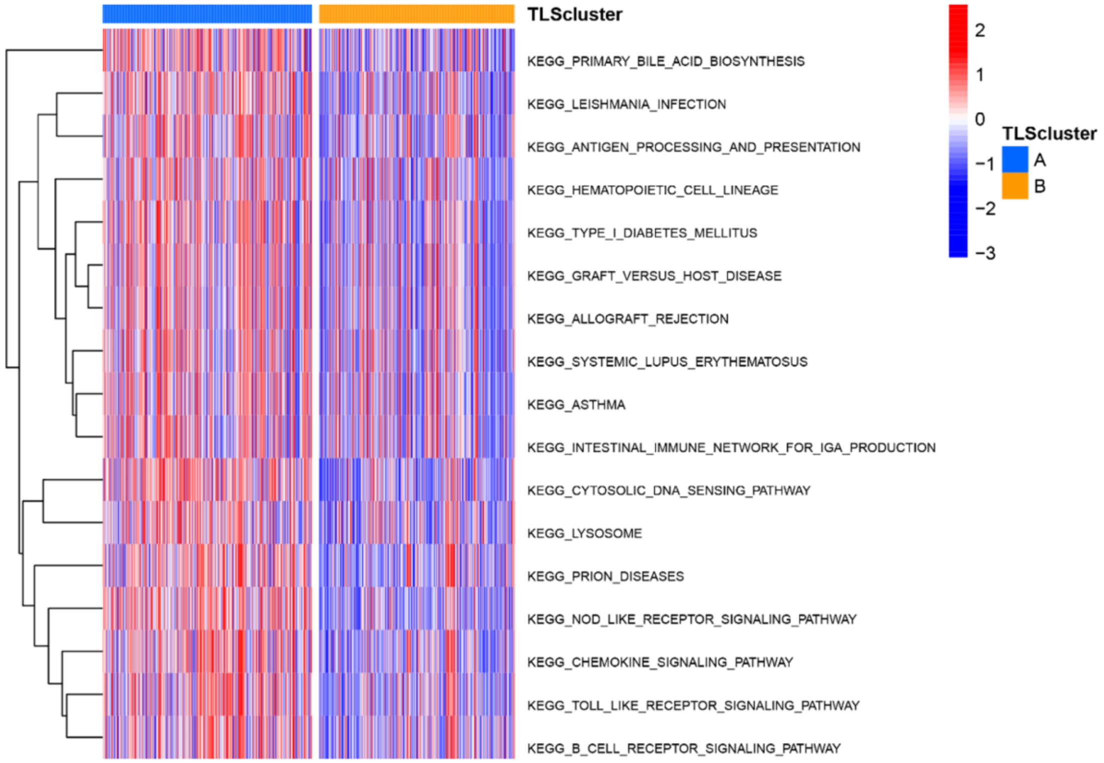

Supplementary Figure 5. The top 20 potential biological functions between subtype A and B in CGGA_cohort1.

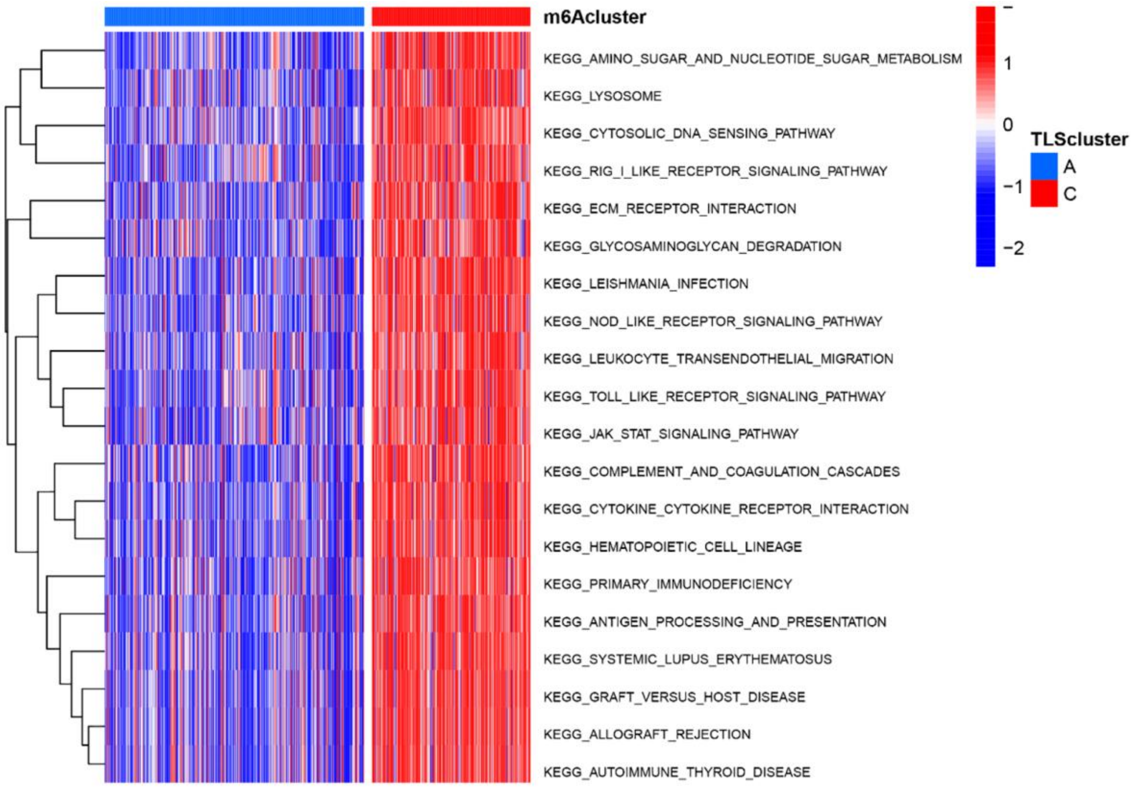

Supplementary Figure 6. The top 20 potential biological functions between subtype A and C in CGGA_cohort1.

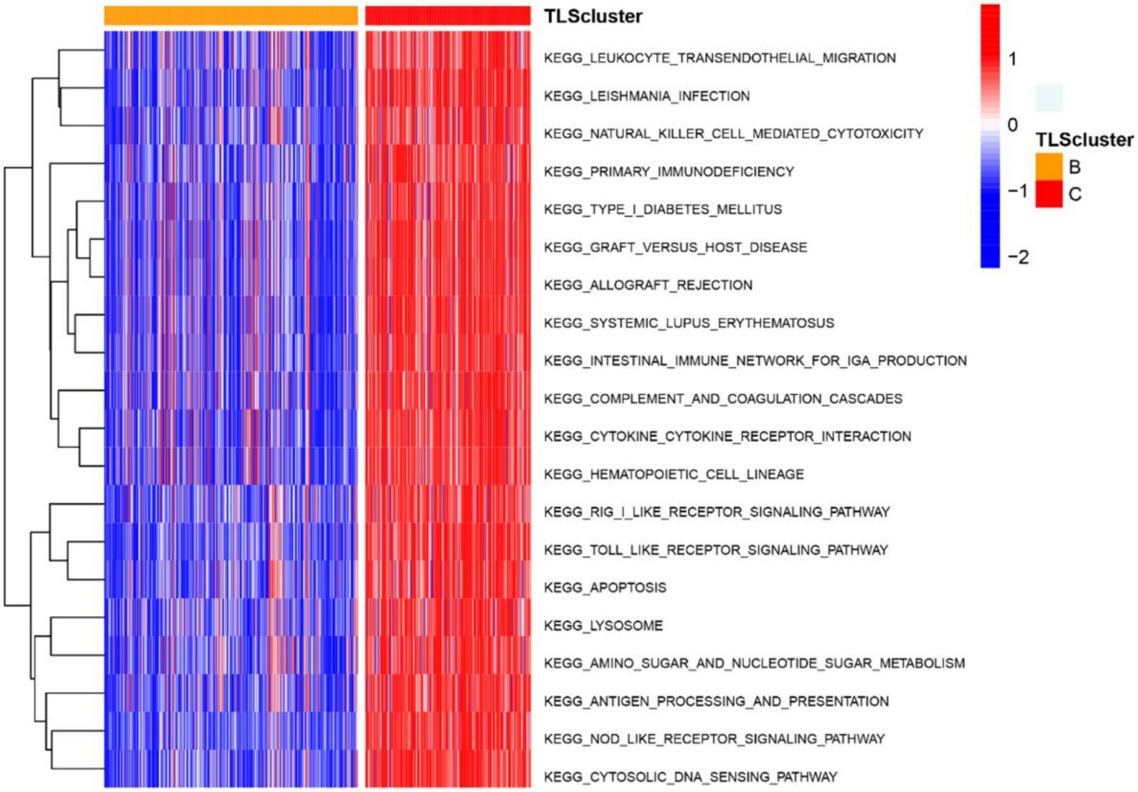

Supplementary Figure 7. The top 20 potential biological functions between subtype B and C in CGGA_cohort1.

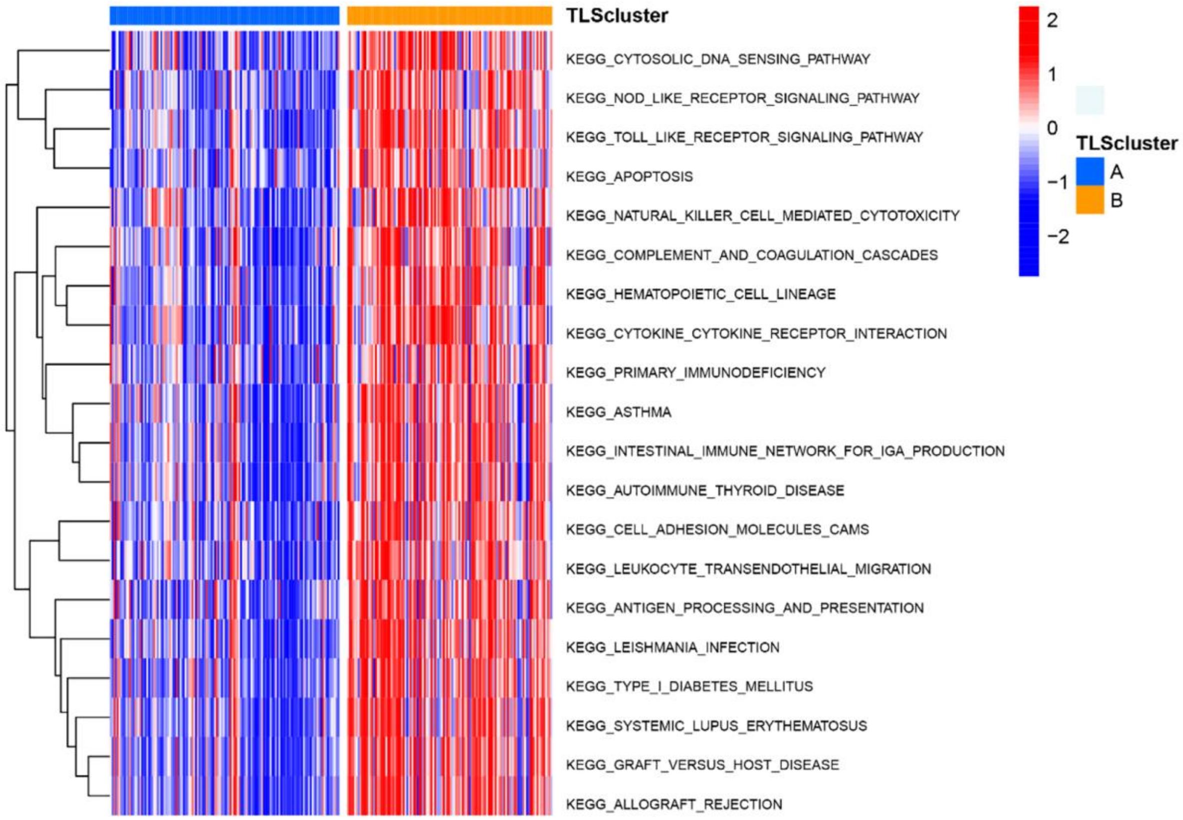

Supplementary Figure 8. The top 20 potential biological functions between subtype A and B in CGGA_cohort2.

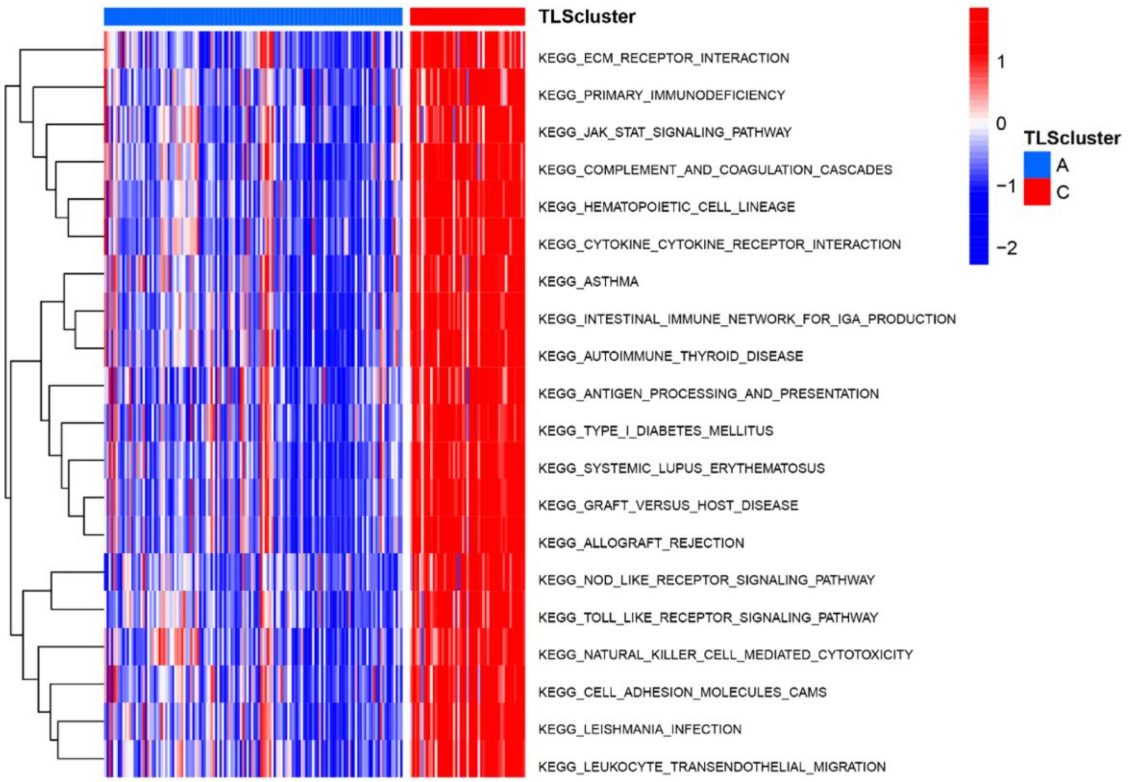

Supplementary Figure 9. The top 20 potential biological functions between subtype A and C in CGGA_cohort2.

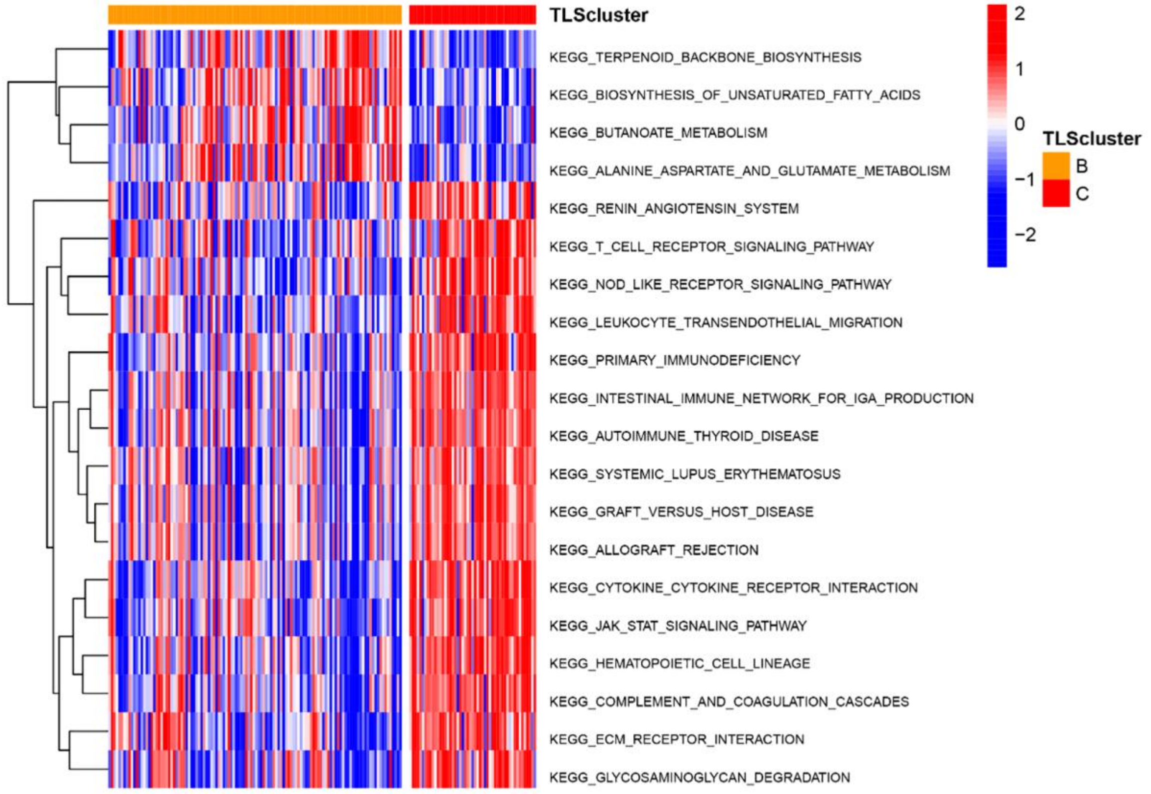

Supplementary Figure 10. The top 20 potential biological functions between subtype B and C in CGGA_cohort2.

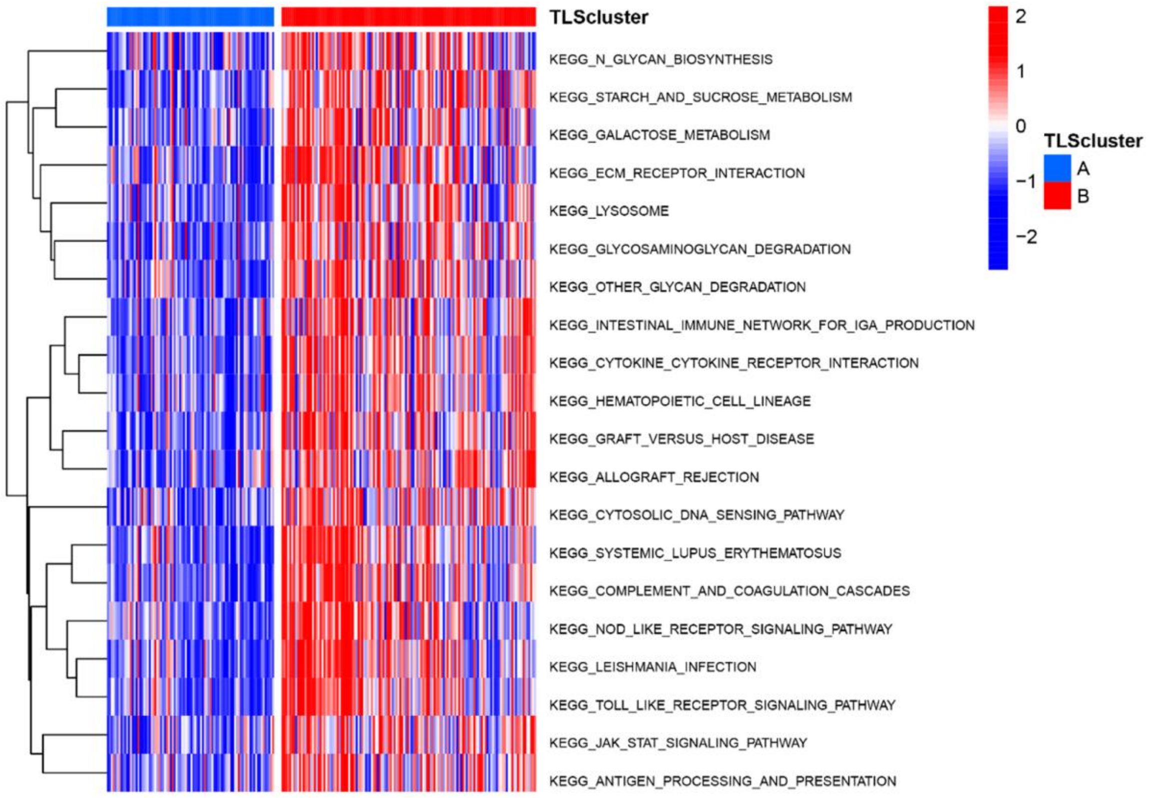

Supplementary Figure 11. The top 20 potential biological functions between subtype A and B in GSE16011.

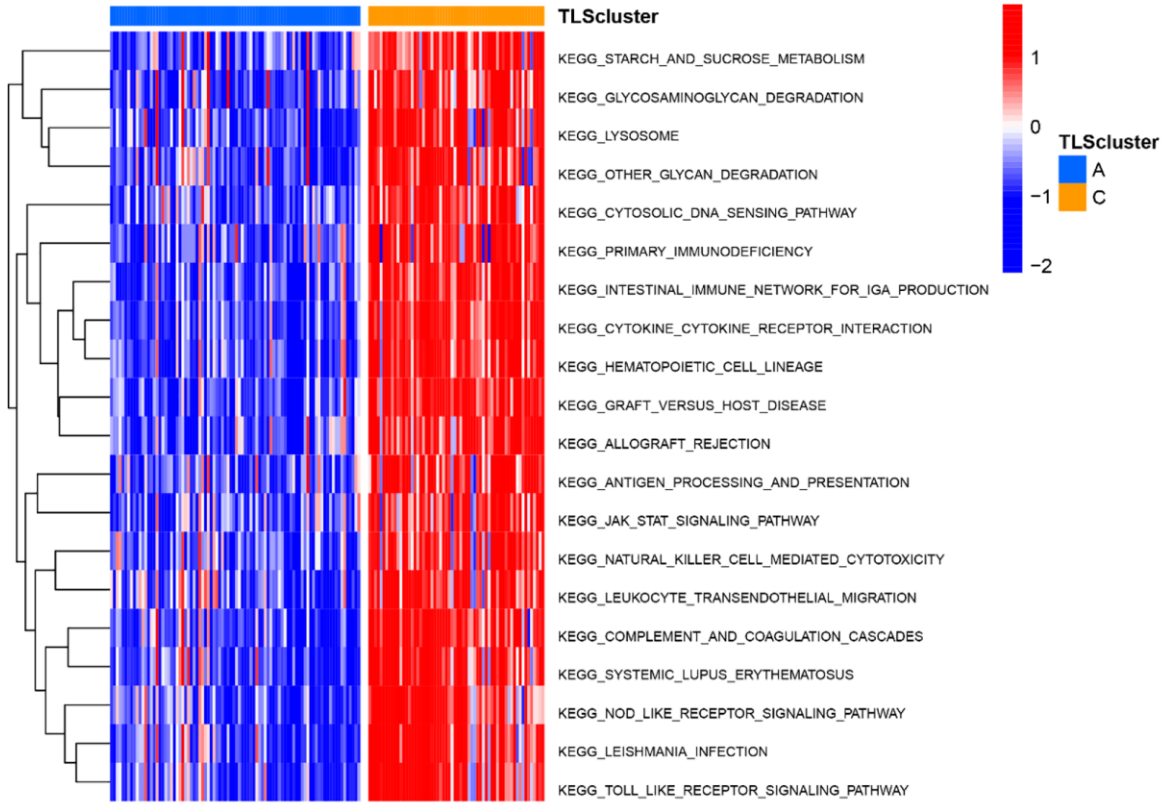

Supplementary Figure 12. The top 20 potential biological functions between subtype A and C in GSE16011.

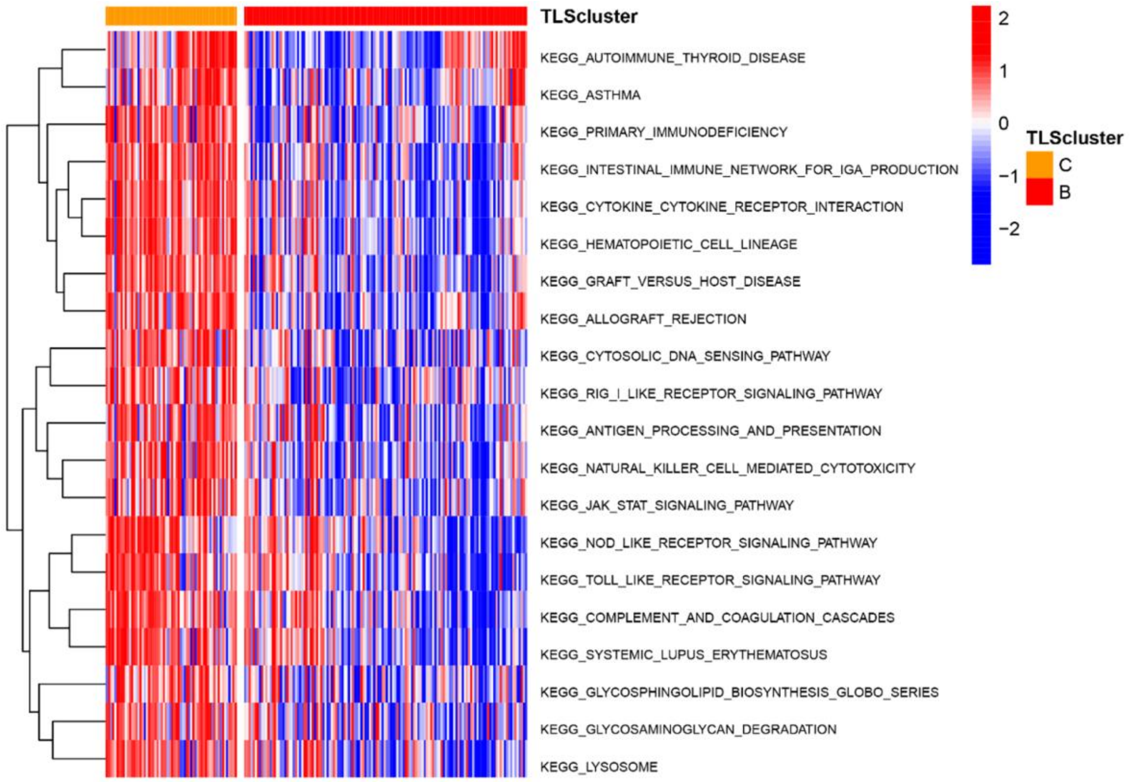

Supplementary Figure 13. The top 20 potential biological functions between subtype B and C in GSE16011.

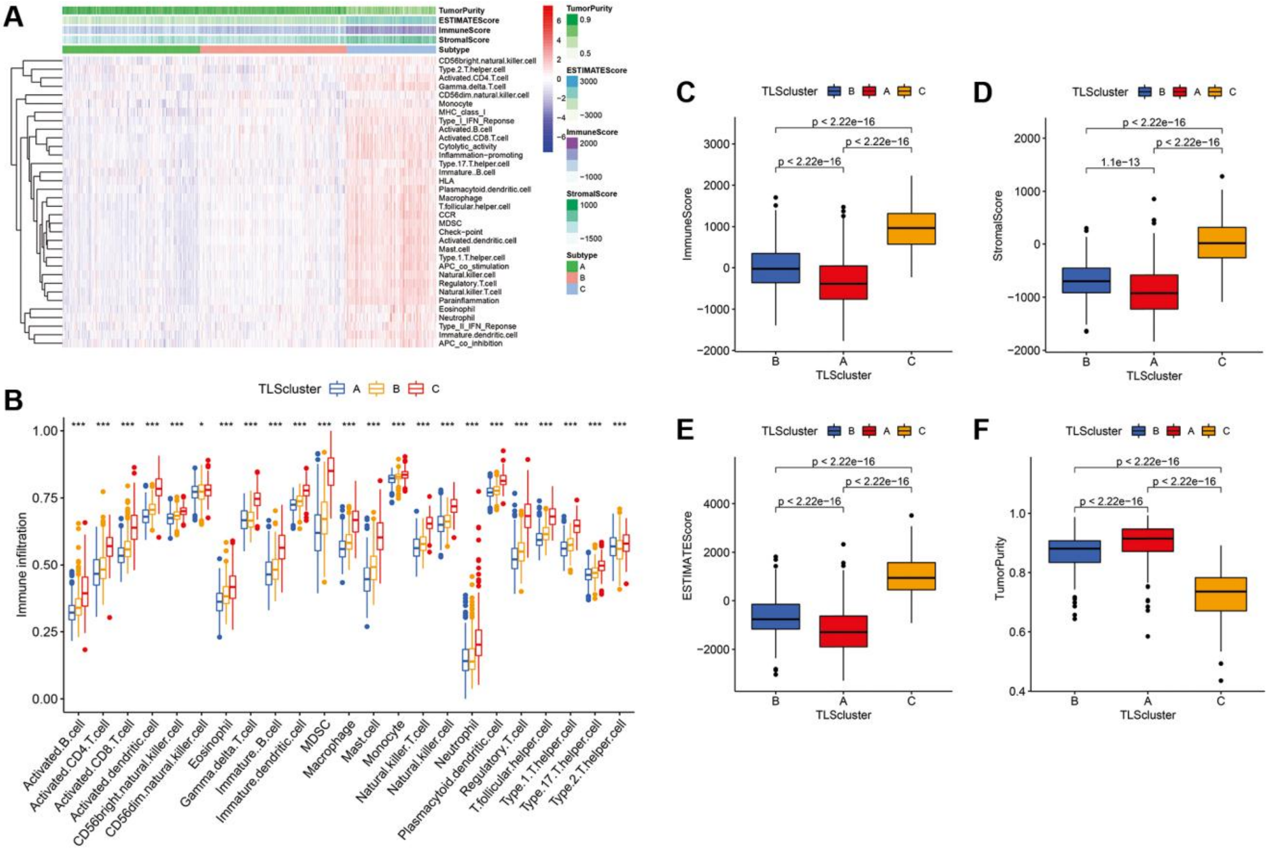

Supplementary Figure 14. Immune infiltration and tumor microenvironment of three metabolic subtypes in CGGA_cohort1. (**A**) Heatmap of TLS subtypes associated with immune infiltration and immune function. (**B**) The signature of 23 immune cell among TLS subtypes. (**C**–**F**) tumor microenvironment of TLS subtypes. C subtype had higher immune, stromal, and ESTIMATE scores compared with the scores of the A and B subtypes; however, tumor purity was lower.

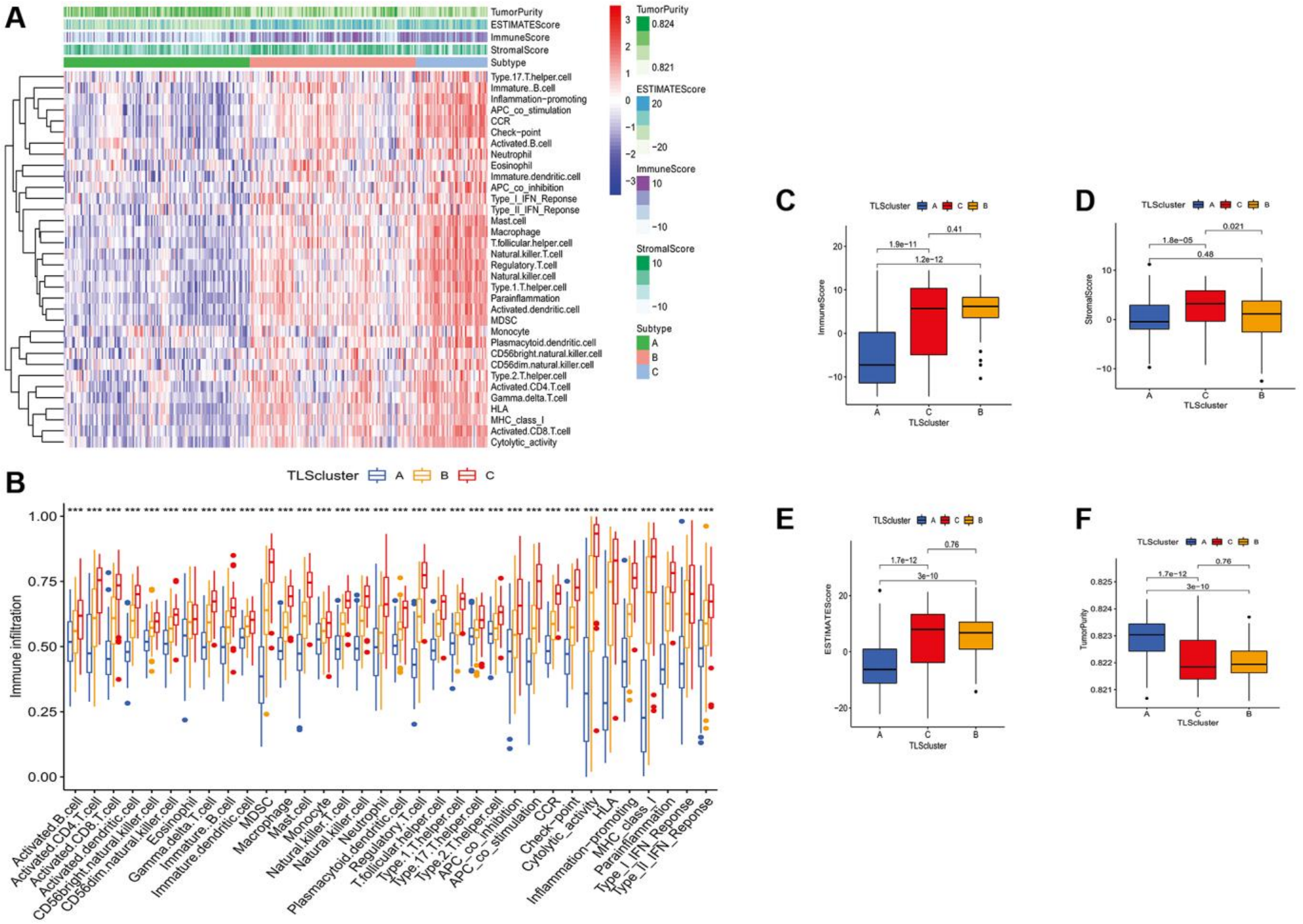

Supplementary Figure 15. Immune infiltration and tumor microenvironment of three metabolic subtypes in CGGA_cohort2. (**A**) Heatmap of TLS subtypes associated with immune infiltration and immune function. (**B**) The signature of 23 immune cell among TLS subtypes. (**C**–**F**) tumor microenvironment of TLS subtypes. C subtype had higher immune, stromal, and ESTIMATE scores compared with the scores of the A and B subtypes; however, tumor purity was lower.

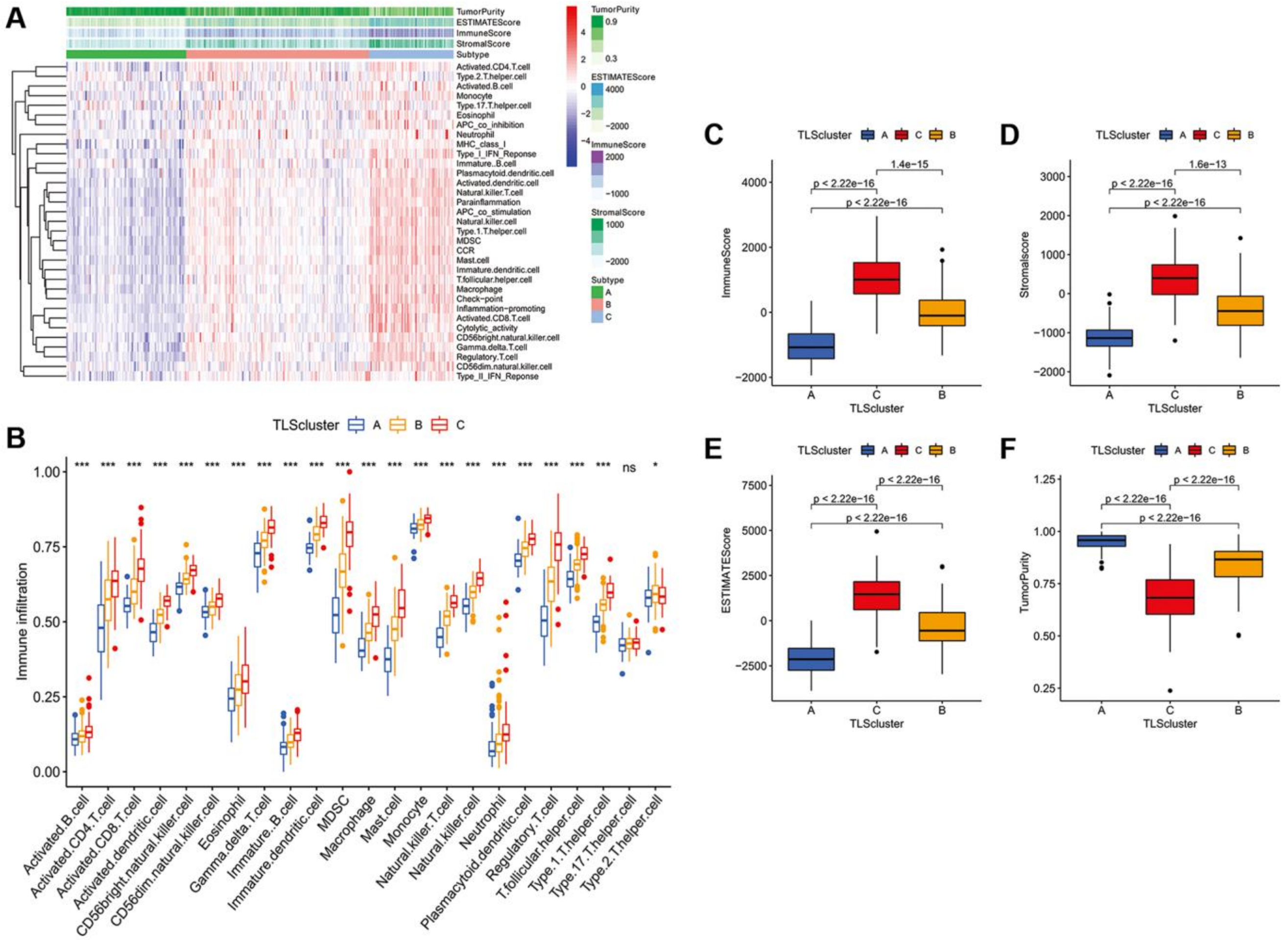

Supplementary Figure 16. Immune infiltration and tumor microenvironment of three metabolic subtypes in GSE16011. (**A**) Heatmap of TLS subtypes associated with immune infiltration and immune function. (**B**) The signature of 23 immune cell among TLS subtypes. (**C**–**F**) tumor microenvironment of TLS subtypes. C subtype had higher immune, stromal, and ESTIMATE scores compared with the scores of the A and B subtypes; however, tumor purity was lower.

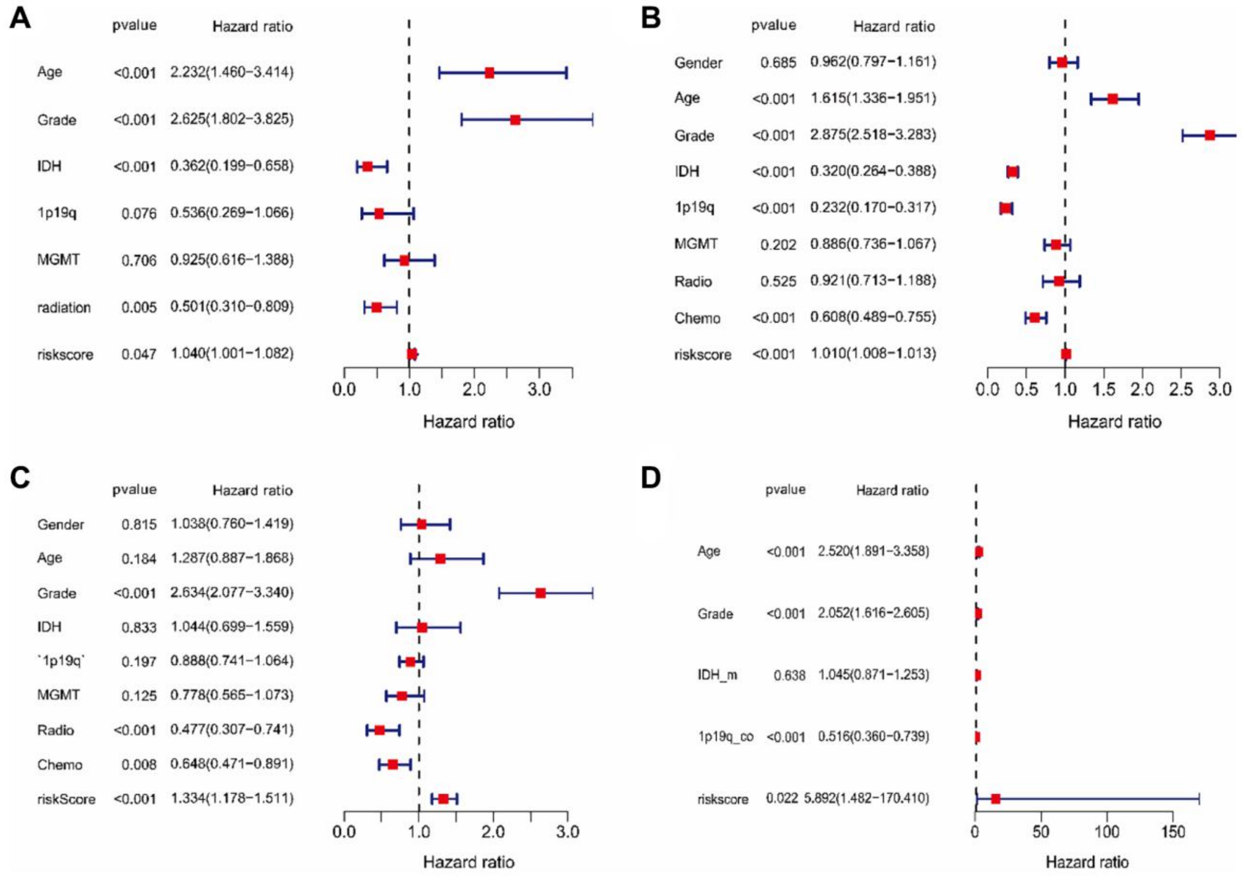

Supplementary Figure 17. Multivariate Cox regression analysis of riskscore in TCGA cohort (**A**), CGGA_cohort1 (**B**), CGGA_cohort2 (**C**) and GSE16011 (**D**).
