# Supplementary material for: Tertiary lymphoid structure stratifies glioma into three distinct tumor subtypes
Source: Aging (Albany NY). 2021 Dec 26;13(24):26063–94. doi: 10.18632/aging.203798 (PMC8751592; doi:10.18632/aging.203798)
Supplement: Supplementary Tables 1-5 [file aging-13-203798-s002.pdf]

SUPPLEMENTARY TABLES

Supplementary Table 1. IGP was estimated for each TLS subtype in three CGGA_cohort1, CGGA_cohort2, and GSE16011.

TLS subtypes	CGGA_cohort1	CGGA_cohort2	GSE16011
A	0.943	0.921	0.933
B	0.865	0.831	0.81
C	0.789	0.709	0.701

Supplementary Table 2. Clinical characteristics of patients with distinct TLS subtypes in TCGA cohort.

Variables	TLS cluster			*p* value
	A	B	C	
Age(Year)				<0.001
≥55	29	61	70	
<55	165	131	53	
Histology				<0.001
A	29	15	2	
AA	39	35	23	
AO	25	43	7	
AOA	12	17	8	
GBM	1	31	81	
O	88	35	0	
OA	22	16	2	
Grade				<0.001
WHO II	114	67	4	
WHO III	79	94	38	
WHO IV	1	31	81	
IDH				
Mutation	187	126	24	
Wildtype	7	66	99	
1p19				<0.001
Codeletion	67	64	3	
Noncodeletion	127	128	120	
MGMT				<0.001
Methylated	172	148	64	
Unmethylated	22	44	59	
Radiotherapy				<0.001
Yes	102	143	99	
No	92	49	24	
Status				<0.001
Live	174	139	61	
Dead	20	53	62	

Supplementary Table 3. Clinical characteristics of patients with distinct TLS subtypes in CGGA_cohort1.

Variables	TLS cluster			*p* value
	A	B	C	
Age (Year)				
≥55	61	42	77	
<55	312	356	164	
Gender				0.424
Male	211	236	149	
Female	162	162	92	
Histology				<0.001
A	45	43	14	
AA	29	39	25	
AO	29	27	7	
AOA	70	90	18	
GBM	117	102	169	
O	33	23	0	
OA	50	74	8	
Grade				<0.001
WHO II	128	140	22	
WHO III	128	156	50	
WHO IV	117	102	169	
IDH				<0.001
Mutation	213	265	51	
Wildtype	143	103	186	
NA	17	30	4	
1p19				<0.001
Codeletion	98	110	3	
Noncodeletion	209	282	236	
NA	66	6	2	
MGMT				0.015
Methylated	191	186	96	
Unmethylated	120	144	109	
NA	62	68	40	
Radiotherapy				<0.001
Yes	280	297	188	
No	65	68	28	
NA	28	33	25	
Chemotherapy				<0.001
Yes	228	235	169	
No	105	123	45	
NA	40	40	27	
Status				<0.001
Live	167	182	48	
Dead	176	190	169	
NA	30	26	24	

Supplementary Table 4. Clinical characteristics of patients with distinct TLS subtypes in CGGA_cohort2.

Variables	TLS cluster			*p* value
	A	B	C	
Age (Year)				0.009
≥55	20	16	16	
<55	112	101	33	
Gender				0.183
Male	72	71	34	
Female	60	46	15	
Histology				<0.001
A	37	41	2	
AA	19	13	5	
AO	6	7	2	
AOA	3	2	0	
GBM	36	38	40	
O	20	3	0	
OA	11	3	0	
Grade				<0.001
WHO II	68	47	2	
WHO III	28	22	7	
WHO IV	2	7	40	
IDH				<0.001
Mutation	77	51	6	
Wildtype	54	66	42	
NA	1	0	1	
1p19				<0.001
Codeletion	13	2	1	
Noncodeletion	33	20	23	
NA	86	95	25	
MGMT				0.04
Methylated	53	29	16	
Unmethylated	71	81	33	
NA	8	7	0	
Radiotherapy				0.012
Yes	105	97	3	
No	21	17	7	
NA	6	3	8	
Chemotherapy				0.163
Yes	56	55	21	
No	64	58	21	
NA	12	4	7	
Status				0.016
Live	54	39	7	
Dead	71	73	40	
NA	7	40	2	

Supplementary Table 5. Clinical characteristics of patients with distinct TLS subtypes in GSE16011.

**Variables**	**TLS cluster**			
	**A**	**B**	**C**	***p* value**
Age (Year)				0.001
≥55	20	51	34	
<55	60	80	26	
Grade				<0.001
WHO I	2	4	1	
WHO II	15	8	1	
WHO III	36	39	10	
WHO IV	27	80	48	
IDH				0.007
Mutation	34	35	12	
Wildtype	28	68	39	
NA	18	28	9	
Status				0.884
Live	10	16	6	
Dead	70	115	54	
