# Supplementary material for: Tertiary lymphoid structure stratifies glioma into three distinct tumor subtypes
Source: Aging (Albany NY). 2021 Dec 26;13(24):26063–94. doi: 10.18632/aging.203798 (PMC8751592; doi:10.18632/aging.203798)
Supplement: Supplementary Table 6 [file aging-13-203798-s003.docx]

**Supplementary Table 6. Association of TLS subtype and drug sensitivity.**

| Drug | IC50 | | |
| --- | --- | --- | --- |
|  | A VS B | A VS C | B VS C |
| A.443654 | A | A | B |
| A.770041 | A | A | B |
| ABT.263 | NS | C | C |
| ABT.888 | NS | A | B |
| AG.014699 | B | C | C |
| AICAR | NS | C | C |
| AKT.inhibitor.VIII | B | NS | NS |
| AMG.706 | NS | NS | NS |
| AP.24534 | B | NS | B |
| AS601245 | B | NS | B |
| ATRA | B | NS | B |
| AUY922 | A | A | B |
| Axitinib | C | B | A |
| AZ628 | A | A | B |
| AZD.0530 | NS | C | C |
| AZD6244 | A | A | B |
| AZD.2281 | A | A | B |
| AZD6482 | NS | A | B |
| AZD7762 | NS | A | B |
| AZD8055 | NS | C | C |
| BAY.61.3606 | B | C | C |
| Bexarotene | NS | A | B |
| BI.2536 | A | A | B |
| BI.D1870 | A | A | B |
| BIBW2992 | NS | A | B |
| Bicalutamide | NS | A | B |
| BIRB.0796 | B | C | C |
| Bleomycin | NS | A | B |
| BMS.509744 | A | A | B |
| BMS.536924 | A | A | B |
| BMS.708163 | NS | C | C |
| BMS.754807 | B | C | C |
| Bortezomib | A | A | B |
| Bosutinib | B | C | NS |
| Bryostatin.1 | A | A | B |
| BX.795 | A | A | B |
| Camptothecin | NS | A | B |
| CCT007093 | B | C | C |
| CCT018159 | NS | A | B |
| CEP.701 | NS | NS | NS |
| CGP.60474 | A | A | B |
| CGP.082996 | A | A | B |
| CHIR.99021 | NS | NS | NS |
| CI.1040 | A | A | B |
| Cisplatin | NS | A | B |
| CMK | A | A | B |
| Cyclopamine | A | A | B |
| Cytarabine | NS | A | B |
| Dasatinib | A | A | B |
| DMOG | NS | NS | NS |
| Docetaxel | A | A | B |
| Doxorubicin | NS | A | B |
| EHT.1864 | B | C | C |
| Elesclomol | B | C | C |
| Embelin | NS | A | B |
| Epothilone.B | NS | NS | B |
| Erlotinib | NS | A | B |
| Etoposide | NS | A | B |
| FH535 | B | C | C |
| FTI.277 | NS | A | B |
| GDC.0449 | NS | A | B |
| GDC0941 | NS | NS | NS |
| Gefitinib | NS | C | NS |
| Gemcitabine | A | A | B |
| GNF.2 | A | A | B |
| GSK269962A | A | A | B |
| GW.441756 | B | C | C |
| GW843682X | A | A | B |
| Imatinib | B | C | C |
| IPA. | NS | C | C |
| JNJ.26854165 | A | A | B |
| JNK.9L | B | C | NS |
| JNK.Inhibitor.VIII | A | A | B |
| JW.7.52.1 | A | A | B |
| KIN001.135 | A | A | NS |
| KU.55933 | A | A | B |
| Lapatinib | A | A | B |
| Lenalidomide | B | C | C |
| LFM.A13 | B | C | C |
| Metformin | A | A | B |
| Methotrexate | NS | A | B |
| MG.132 | A | A | B |
| Midostaurin | NS | A | B |
| Mitomycin.C | A | A | B |
| MK.2206 | B | A | B |
| MS.275 | A | A | B |
| Nilotinib | NS | C | C |
| NSC.87877 | NS | A | B |
| NU.7441 | A | A | B |
| Nutlin.3a | NS | A | B |
| NVP.BEZ235 | A | A | B |
| NVP.TAE684 | A | A | B |
| Obatoclax.Mesylate | NS | A | B |
| OSI.906 | B | C | C |
| PAC.1 | NS | NS | NS |
| Paclitaxel | A | A | B |
| Parthenolide | A | A | B |
| PD.173074 | B | C | C |
| PD.0325901 | A | A | B |
| PD.0332991 | B | C | C |
| PF.562271 | B | C | C |
| PF.4708671 | NS | NS | NS |
| PHA.665752 | A | A | B |
| PLX4720 | NS | A | B |
| Pyrimethamine | NS | A | B |
| QS11 | B | C | C |
| Rapamycin | A | A | B |
| RDEA119 | A | A | B |
| RO.3306 | NS | A | B |
| Roscovitine | A | A | B |
| S.Trityl.L.cysteine | A | A | B |
| Salubrinal | NS | A | B |
| SB.216763 | NS | A | B |
| Shikonin | B | A | B |
| SL.0101.1 | A | A | NS |
| Sorafenib | A | A | B |
| Sunitinib | A | A | B |
| Temsirolimus | A | A | B |
| Thapsigargin | B | C | C |
| Tipifarnib | B | A | B |
| TW.37 senstivity | A | A | B |
| Vinblastine | NS | A | B |
| Vinorelbine | B | C | C |
| Vorinostat | B | C | C |
| VX.680 | A | A | B |
| VX.702 | B | C | C |
| WH.4.023 | A | A | B |
| WO2009093972 | A | A | B |
| WZ.1.84 | A | A | B |
| X17.AAG | A | A | B |
| X681640 | NS | C | C |
| XMD8.85 | A | A | B |
| Z.LLNle.CHO | A | A | B |
