# Supplementary material for: Tertiary lymphoid structure stratifies glioma into three distinct tumor subtypes
Source: Aging (Albany NY). 2021 Dec 26;13(24):26063–94. doi: 10.18632/aging.203798 (PMC8751592; doi:10.18632/aging.203798)
Supplement: Supplementary Table 7 [file aging-13-203798-s004.pdf]

SUPPLEMENTARY TABLES

Supplementary Table 7. The genes used to define the immune cell population.

Immune cell	Gene										
Activated.B.cell	ADAM28	CD180	CD79B	BLK	CD19	MS4A1	TNFRSF17	IGHM	GNG7	MICAL3	SPIB
	HLA-DOB	IGKC	PNOC	FCRL2	BACH2	CR2	TCL1A	AKNA	ARHGAP25	CCL21	CD27
	CD38	CLEC17A	CLEC9A	CLECL1							
Activated.CD4.T.cell	AIM2	BIRC3	BRIP1	CCL20	CCL4	CCL5	CCNB1	CCR7	DUSP2	ESCO2	
	ETS1	EXO1	EXOC6	IARS	ITK	KIF11	KNTC1	NUF2	PRC1	PSAT1	RGS1
	RTKN2	SAMSN1	SELL	TRAT1							
Activated.CD8.T.cell	ADRM1	AHSA1	C1GALT1C1	CCT6B	CD37	CD3D	CD3E	CD3G	CD69	CD8A	CETN3
	GPT2	GZMA	GZMH	GZMK	IL2RB	LCK	MPZL1	NKG7	PIK3IP1	PTRH2	TIMM13
	CSE1L	GEMIN6	GNLY	ZAP70							
Activated.dendritic.cell	ABCD1	C1QC	CAPG	CCL3L3	CD207	CD302	ATP5B	ATP5L	ATP6V1A	BCL2L1	C1QB
	SNURF	SPCS3	CCNA1	CEACAM8	NOS2	SRA1	TNFRSF6B	TREM1	TREML1	RHOA	SLC25A37
	TNFSF14	TREML4	VNN2	XPO6	CLEC4C	TNFAIP2	UBD	ACTR3	RAB1A	SLA	HLA-DQA2
	SIGLEC5	SLAMF9									
CD56bright.natural.killer.cell	ABAT	C11orf75	C5orf15	CDHR1	DCAF12	DYNLL1	GPR137B	HCP5	HDGFRP2	KRT86	MLST8
	ELMOD3	ENTPD5	FAM119A	FAM179A	CLIC2	COX7A2L	CREB3L4	CSF1	CSNK2A2	CSTA	CSTB
	CTPS	CTSD	FST	GATA2	GMPR	HDC	HEY1	HOXA1	HS2ST1	HS3ST1	BCL11B
	CDH3	MYL6B	NAA16	ClQA	ClQB	CYP27B1	EIF3M				
CD56dim.natural.killer.cell	CYP27A1	DDX55	DYRK2	RPL37A	NOTCH3	AKR7A3	GPRC5C	GRIN1	HLA-E	PORCN	PSMC4
	UPP1	IL21R	KIR2DS1	KIR2DS2	KIR2DS5						
Eosinophil	GIPR	KRT18P50	LRMP	FOSB	RRP12	GPR183	NR4A3	ST3GAL6	DEPDC5	PDE6C	PKD2L2
	GPR65	IL5RA	P2RY14	DACH1	DAPK2	EMR3					
Gamma.delta.T.cell	ACP5	AQP9	BTN3A2	C1orf54	CARD8	CCL18	CD209	CD33	CD36	CDK5	IL10RB
	KLRF1	LGALS1	MAPK7	KLHL7	KRT80	LAMC1	LCORL	LMNB1	MEIS3P1	MPL	FABP1
	FABP5	FADD	MFAP3L	MINPP1	RPS24	RPS7	RPS9	DBNL	CCL13		
Immature..B.cell	CD22	CYBB	FAM129C	FCRL1	FCRL3	FCRL5	FCRLA	HDAC9	HLA-DQA1	HVCN1	KIAA0226
	NCF1	NCF1B	P2RY10	SP100	TXNIP	STAP1	TAGAP	ZCCHC2			
Immature.dendritic.cell	ACADM	AHCYL1	ALDH1A2	ALDH3A2	ALDH9A1	ALOX15	AMT	ARL1	ATIC	ATP5A1	CAPZA1
	LILRA5	RDX	RRAGD	TACSTD2	INPP5F	RAB38	PLAU	CSF3R	SLC18A2	AMPD2	CLTB
	C1orf162										
MDSC	CCR2	CD14	CD2	CD86	CXCR4	FCGR2A	FCGR2B	FCGR3A	FERMT3	GPSM3	IL18BP
	IL4R	ITGAL	ITGAM	PARVG	PSAP	PTGER2	PTGES2	S100A8	S100A9		
Macrophage	AIF1	CCL1	CCL14	CCL23	CCL26	CD300LB	CNR1	CNR2	EIF1	EIF4A1	FPR1
	FPR2	FRAT2	GPR27	GPR77	RNASE2	MS4A2	BASP1	IGSF6	HK3	VNN1	FES
	NPL	FZD2	FAM198B	HNMT	SLC15A3	CD4	TXNDC3	FRMD4A	CRYBB1	HRH1	WNT5B
Mast.cell	ADAMTS3	CPA3	CMA1	CTSG	ARHGAP15	CPM	FCN1	FTL	HSPA6	ITGA9	RNASE3
	S100A4	SIGLEC8	SLC6A4	PTGS2	EGR3	PILRA					
Monocyte	ASGR2	CFP	ASGR1	CD1D	UPK3A	ACTG1	ANXA5	ATP6V1B2	CFL1	DAZAP2	CTBS
	EMR4P	HIVEP2	MARCKSL1	MBP	MMP15	PNPLA6	TMBIM6	PQBP1	TEX264	IKZF1	
Natural.killer.T.cell	BTN2A2	CD101	CD109	CNPY3	CNPY4	CREB1	CRTC2	CRTC3	CSF2	KLRC1	FUT4
	ICAM2	IL32	LAMP2	LILRB5	KLRG1	HSPA4	HSPB6	ISM2	ITIH2	KDM4C	KIR2DS4
	KIRREL3	SDCBP	NFATC2IP	MICB	KIR2DL1	KIR2DL3	KIR3DL1	KIR3DL2	NCR1	FOSL1	TSLP
	SLC7A7	SPP1	TREM2	UBASH3A	YBX2	CCDC88A	CLEC1A	THBD	PDPN	VCAM1	EMR1
Natural.killer.cell	AKT3	AXL	BST2	CDH2	CRTAM	CSF2RA	CTSZ	CXCL1	CYTH1	DAXX	DGKH
	DLL4	DPYD	ERBB3	F11R	FAM27A	FAM49A	FASLG	FCGR1A	FN1	FSTL1	FUCA1

	GBP3	GLS2	GRB2	LST1	BCL2	CDC5L	FGF18	FUT5	FZR1	GAGE2	IGFBP5
	KANK2	LDB3									
Neutrophil	CREB5	CDA	CHST15	S100A12	APOBEC3A	CASP5	MMP25	HAL	C1orf183	FFAR2	MAK
	CXCR1	STEAP4	MGAM	BTNL8	CXCR2	TNFRSF10C	VNN3				
Plasmacytoid.dendritic.cell	CBX6	DAB2	DDX17	HIGD1A	IDH3A	IL3RA	MAGED1	NUCB2	OFD1	OGT	PDIA4
	SERTAD2	SIRPA	TMED2	ENG	FCAR	IGF1	ITGA2B	GABARAP	GPX1	KRT23	PROK2
	RALB	RETNLB	RNF141	SEC14L1	SEPX1	EMP3	CD300LF	ABTB1	KLHL21	PHRF1	
Regulatory.T.cell	CCL3L1	CD72	CLEC5A	FOXP3	ITGA4	L1CAM	LIPA	LRP1	LRRC42	MARCO	MMP12
	MNDA	MRC1	MS4A6A	PELO	PLEK	PRSS23	PTGIR	ST8SIA4	STAB1		
T.follicular.helper.cell	B3GAT1	CDK5R1	PDCD1	BCL6	CD200	CD83	CD84	FGF2	GPR18	CEBPA	CECR1
	CLEC10A	CLEC4A	CSF1R	CTSS	DMN	DPP4	LRRC32	MC5R	MICA	NCAM1	NCR2
	NRP1	PDCD1LG2	PDCD6	PRDX1	RAE1	RAET1E	SIGLEC7	SIGLEC9	TYRO3	CHST12	CLIC3
	IVNS1ABP	KIR2DL2	LGMN								
Type.1.T.helper.cell	CD70	TBX21	ADAM8	AHCYL2	ALCAM	B3GALNT1	BBS12	BST1	CD151	CD47	CD48
	CD52	CD53	CD59	CD6	CD68	CD7	CD96	CFHR3	CHRM3	CLEC7A	COL23A1
	COL4A4	COL5A3	DAB1	DLEU7	DOC2B	EMP1	F12	FURIN	GAB3	GATM	GFPT2
	GPR25	GREM2	HAVCR1	HSD11B1	HUNK	IGF2	RCSD1	RYR1	SAV1	SELE	SELP
	SH3KBP1	SIT1	SLC35B3	SIGLEC10	SKAP1	THUMPD2	TIGIT	ZEB2	ENC1	FAM134B	FBXO30
	FCGR2C	STAC	LTC4S	MAN1B1	MDH1	MMD	RGS16	IL12A	P2RX5	CD97	ITGB4
	ICAM3	METRNL	TNFRSF1A	IRF1	HTR2B	CALD1	MOCOS	TRAF3IP2	TLR8	TRAF1	DUSP14
Type.17.T.helper.cell	IL17A	IL17RA	C2CD4A	C2CD4B	CA2	CCDC65	CEACAM3	IL17C	IL17F	IL17RC	IL17RE
	IL23A	ILDR1	LONRF3	SH2D6	TNIP2	ABCA1	ABCB1	ADAMTS12	ANK1	ANKRD22	B3GALT2
	CAMTA1	CCR9	CD40	GPR44	IFT80						
Type.2.T.helper.cell	ASB2	CSRP2	DAPK1	DLC1	DNAJC12	DUSP6	GNAI1	LAMP3	NRP2	OSBPL1A	PDE4B
	PHLDA1	PLA2G4A	RAB27B	RBMS3	RNF125	TMPRSS3	GATA3	BIRC5	CDC25C	CDC7	CENPF
	CXCR6	DHFR	EVI5	GSTA4	HELLS	IL26	LAIR2				
